# Update on the third international stroke trial (IST-3) of thrombolysis for acute ischaemic stroke and baseline features of the 3035 patients recruited

**DOI:** 10.1186/1745-6215-12-252

**Published:** 2011-11-30

**Authors:** Peter Sandercock, Richard Lindley, Joanna Wardlaw, Martin Dennis, Karen Innes, Geoff Cohen, Will Whiteley, David Perry, Vera Soosay, David Buchanan, Graham Venables, Anna Czlonkowska, Adam Kobayashi, Eivind Berge, Karsten Bruins Slot, Veronica Murray, Andre Peeters, Graeme J Hankey, Karl Matz, Michael Brainin, Stefano Ricci, Teresa A Cantisani, Gordon Gubitz, Stephen J Phillips, Arauz Antonio, Manuel Correia, Phillippe Lyrer, Ingrid Kane, Erik Lundstrom

**Affiliations:** 1 The IST-3 Co-ordinating Centre, Neurosciences Trial Unit, Bramwell Dott Building, Western General Hospital, Crewe Road, Edinburgh EH4 2XU, UK; 2 Discipline of Medicine, Sydney Medical School - Westmead and The George Institute for Global Health, University of Sydney, NSW 2006, Australia; 3 Neurology Department, Sheffield Teaching Hospitals NHS Foundation Trust, Royal Hallamshire Hospital, Glossop Road, Sheffield S10 2JF. UK; 4 2nd Department of Neurology, Institute of Psychiatry and Neurology, Sobieskiego Str. 9, 02- 957 Warsaw, and Department of Experimental and Clinical Pharmacology, Medical University of Warsaw. Krakowskie Przedmiescie Str. 26/28, 00-927 Warsaw, Poland; 5 Department of Internal Medicine, Oslo University Hospital, Ullevål, Kirkeveien 166, NO-0407 Oslo, Norway; 6 Department of Clinical Sciences, Karolinska Institutet, Danderyd Hospital, SE-182 88 Stockholm, Sweden; 7 Service de neurologie, Cliniques universitaires Saint-Luc, Avenue Hippocrate 10, 1200 Bruxelles, Belgium; 8 Department of Neurology, Royal Perth Hospital, Wellington Street, GPO Box X2213, Perth, Western Australia, 6001, Australia; 9 Neurologische Abteilung, Landesklinikum Donauregion Tulln, Alter Ziegelweg 10, 3430 Tulln, Austria; 10 UO Neurologia, ASL 1, Ospedale, Via Engels, 06012 Citta' di Castello PG, Italy; 11 S C di Neurofisiopatologia, Azienda Ospedaliera di Perugia, Italy; 12 Division of Neurology, Dalhousie University and Queen Elizabeth II Health Sciences Centre, Halifax Infirmary, 1796 Summer Street, Halifax, Nova Scotia B3H 3A7, Canada; 13 Instituto Nacional de Neurologia, Insurgentes sur 3877, La Fama, 14269 Mexico DF, Mexico; 14 Neurology Department, Hospital Geral de Santo Antonio, Largo Prof Abel Salazar, 4050 Porto, Portugal; 15 Department of Neurology, University Hospital Basel, Petersgraben 4, CH-4031 Basel, Switzerland; 16 Stroke Unit, Royal Sussex County Hospital, Eastern Road, Brighton, East Sussex, BN2 5BE, UK; 17 Neurology, Institute of Neuroscience, Uppsala University Hospital, Ing 85, 2 tr, SE-751 85 Uppsala, Sweden

## Abstract

**Background:**

Intravenous recombinant tissue plasminogen activator (rtPA) is approved in Europe for use in patients with acute ischaemic stroke who meet strictly defined criteria. IST-3 sought to improve the external validity and precision of the estimates of the overall treatment effects (efficacy and safety) of rtPA in acute ischaemic stroke, and to determine whether a wider range of patients might benefit.

**Design:**

International, multi-centre, prospective, randomized, open, blinded endpoint (PROBE) trial of intravenous rtPA in acute ischaemic stroke. Suitable patients had to be assessed and able to start treatment within 6 hours of developing symptoms, and brain imaging must have excluded intracranial haemorrhage and stroke mimics.

**Results:**

The initial pilot phase was double blind and then, on 01/08/2003, changed to an open design. Recruitment began on 05/05/2000 and closed on 31/07/2011, by which time 3035 patients had been included, only 61 (2%) of whom met the criteria for the 2003 European approval for thrombolysis. 1617 patients were aged over 80 years at trial entry. The analysis plan will be finalised, without reference to the unblinded data, and published before the trial data are unblinded in early 2012. The main trial results will be presented at the European Stroke Conference in Lisbon in May 2012 with the aim to publish simultaneously in a peer-reviewed journal. The trial result will be presented in the context of an updated Cochrane systematic review. We also intend to include the trial data in an individual patient data meta-analysis of all the relevant randomised trials.

**Conclusion:**

The data from the trial will: improve the external validity and precision of the estimates of the overall treatment effects (efficacy and safety) of iv rtPA in acute ischaemic stroke; provide: new evidence on the balance of risk and benefit of intravenous rtPA among types of patients who do not clearly meet the terms of the current EU approval; and, provide the first large-scale randomised evidence on effects in patients over 80, an age group which had largely been excluded from previous acute stroke trials.

**Trial registration:**

ISRCTN25765518

## Progress with the study and modifications to the design

Planning for the third international stroke trial (IST-3) of thrombolysis for acute ischaemic stroke with intravenous recombinant tissue plasminogen activator (rtPA) began in 1998. At its outset in 2000, the trial was planned as a pragmatic trial which sought to improve the external validity and precision of the estimates of the overall treatment effects (efficacy and safety) of rtPA in acute ischaemic stroke and to determine whether a wider variety of patients than previously thought might benefit from this treatment. After the drug was approved for use in Europe in 2003, the flexible trial eligibility criteria enabled us to continue recruiting patients who did not clearly meet all the criteria set out in the provisional European licence for the drug. With these changes and the need to comply with the increasingly burdensome requirements of research regulation in Europe, some aspects of the trial protocol and its practical procedures have inevitably had to change over the 12 year course of the study. The initial pilot phase of the study, funded by the UK Stroke Association, had a double-blind design, for which Boehringer Ingelheim provided a supply of drug and matching placebo. However, Boehringer Ingelheim did not wish to provide further drug supplies for the expansion phase, funded by The UK Health Foundation, and hence the design of this phase of the study was modified. With appropriate ethical approval, the expansion phase was designed as a prospective, randomised, open blinded endpoint (PROBE) trial and the first patient was recruited in this phase on 1^st ^August 2003. The main phase of the trial was awarded funding by the UK Medical Research Council and began on 1^st ^April 2005. The protocol for the MRC phase of the trial was published in *Trials*[1].. At each stage, relevant amendments were made to the protocol, and consolidated in an updated protocol (version 1.93) which was approved by appropriate authorities and published on the trial website http://www.ist3.com (additional data file 1). When advanced imaging techniques became more widely available in the participating centres, the study group developed a nested imaging study evaluating the role of CT and MR perfusion and angiography (CTP/CTA/MRP/MRA) in patients with acute ischaemic stroke. The cost-effectiveness of thrombolysis is being investigated in a sub-study in the Scandinavian region cohort.

## Sample size/recruitment target

In our 2004 application to the MRC for funding of the main phase of the trial we stated: *'Assuming the same event rate as observed in the feasibility phases, the trial could very reliably detect an absolute difference of 10% in the proportion of patients dead or dependent at 6 months after treatment. With a sample size of 6000 patients, the trial would have > 99.9% power to detect such a difference (alpha = 0.001) and sufficient power to permit reliable analyses of the pre-specified subgroups. A trial of this size could detect a smaller, but still clinically worthwhile net benefit of as little as a 3% absolute difference in the primary outcome (80% power, at alpha = 0.05).' *The application to the MRC for funding was successful, but by the time the grant was activated, two major changes to research regulation came into force: the European Clinical Trials Directive, the UK Clinical Trials Regulations 2004, and, shortly afterwards, the UK Research Governance Framework. These changes led to a large increase in the time taken to recruit and activate new centres and hence significantly reduced the rate of increase in patient recruitment. By 2007, it was clear that the various factors that had delayed recruitment meant that the original target of 6000 was no longer feasible, and we applied for approval to amend the recruitment target and for additional funding to extend the period of recruitment to mid 2011. These changes were summarised in version 1.93 of the protocol (additional document 1) as follows: "*If 3500 patients were recruited, the trial could detect a 4% absolute difference in the primary outcome. A sample size of 1000 patients could detect a 7% absolute difference in the primary outcome, which is consistent with the effect size among patients randomised within 3 hours of stroke in the Cochrane review. **Amendment in the light of recruitment by 2007:**. The Medical Research Council... awarded an extension to funding to permit recruitment to continue until mid 2011, with a revised recruitment target of 3100 which would yield 80% power to detect an absolute difference of 4.7% in the primary outcome.'*

## Recruitment

The pilot (double blind) phase of the trial began when the first patient was randomised on 5^th ^May 2000; sixteen centres enrolled patients. Recruitment continued without interruption into the open expansion phase (first patient in this phase was randomised on 1^st ^August 2003). In April 2005, recruitment continued with the start of the MRC funded main phase. The independent Data Monitoring Committee (DMC) met regularly throughout the trial, in strict confidence, to review the accumulating trial data and any other relevant external data. Their terms of reference were set out in the protocol, and in the light of the evidence available, the DMC did not recommend any change to the protocol or to recruitment into the study. The trial closed to recruitment on 31^st ^July 2011, with a total of 3035 patients from 156 hospitals in 12 countries (Table 1).

**Table 1:** Recruitment: number of centres and patients in each country

	No. of centres	No. of patients	%
UK	75	1447	47.7%
Poland	9	347	11.4%
Italy	21	326	10.7%
Sweden	18	297	9.8%
Norway	11	204	6.7%
Australia	10	179	5.9%
Portugal	4	82	2.7%
Belgium	1	73	2.4%
Austria	3	46	1.5%
Switzerland	2	23	0.8%
Canada	1	8	0.3%
Mexico	1	3	0.1%
Total	156	3035	100%

## Eligibility

The eligibility criteria are set out in full in the protocol, but can be summarised in terms of the 'uncertainty principle'[2-4]. As this was a pragmatic trial, conducted in the ';real world'[5], in order to ensure a wide variety of patients were included, inclusion and exclusion criteria were kept simple [6]. Patients were eligible if: they had symptoms and signs of clinically definite acute stroke; the time of stroke onset was known; treatment could be started within six hours of this onset; and, CT or MRI brain scanning had reliably excluded both intracranial haemorrhage and structural brain lesions which could mimic stroke (e.g cerebral tumour). In addition, if the patient had a clear indication for intravenous thrombolysis with rtPA, they were to be treated in accordance with local guidelines. Equally, if the patient had a clear contraindication to treatment they were not to be entered in the trial. Only if both the clinician and the patient (or a relevant proxy for the patient) felt that the treatment was 'promising but unproven', could the patient be included in the trial after appropriate informed consent. In order to comply with local regulatory and ethical requirements, the form of the consent procedure applied varied somewhat between countries and between centres within each country. Details of the type of consent process applied will be reported separately.

## Baseline data collection and method of treatment allocation

After informed consent had been obtained, the clinician used either a voice-activated telephone or a secure web-based randomisation system to enter a patient in the study. The systems recorded and checked the baseline characteristics of the patients and allocated the treatment only after all baseline data had been entered and passed range and consistency checks. We used a minimisation program to achieve optimum balance for key prognostic factors (see Table 2a), and from January 2006, the minimisation algorithm was additionally stratified by world region and minimised on everything else within world region. Because simple minimisation within centres can lead to alternation of treatment allocation and thus potential loss of allocation concealment, our system also incorporated a degree of random allocation. Patients were allocated to the treatment group that would minimise the difference between the groups with a probability of 0.80. For 244 patients in the initial phase of the trial, the National Institutes of Health Stroke Score (NIHSS) was not measured directly, but was retrospectively estimated by an algorithm (derived from subsequent patients in the trial based on baseline clinical characteristics). In the initial double-blind phase, the clinician was informed of the treatment allocation in the form of a treatment pack number (which might contain active drug or matching placebo). In the open phase, the investigator was informed of the patient's treatment allocation (either thrombolysis with 0.9 mg/kg i.v. rtPA or control). In both phases, the randomisation system informed the local clinician of the dose (of drug or placebo in the double blind phase, or of the drug dose amongst those allocated thrombolysis in the open phase) to be given as a bolus and as infusion, and the patient's unique trial ID number.

**Table 2a:** Clinical characteristics at randomisation used in the minimisation algorithm

		**No**.	(%)
Region^1^	NW Europe (UK, Austria, Belgium, Switzerland)	1589	(52%)
	Scandinavia (Norway, Sweden)	501	(17%)
	Australasia	179	(6%)
	Southern Europe (Italy, Portugal)	408	(13%)
	Eastern Europe (Poland)	347	(11%)
	Americas (Canada, Mexico)	11	(0%)

Sex	Female	1570	(52%)
	Male	1465	(48%)

Age	< = 70 years	697	(23%)
	> 70 years	2338	(77%)

Age (more detail)	18-50	127	(4%)
	51-60	203	(7%)
	61-70	367	(12%)
	71-80	721	(24%)
	81-90	1407	(46%)
	over 90	210	(7%)

NIHSS^2^	0 to 5	612	(20%)
	6 to 10	852	(28%)
	11 to 15	601	(20%)
	16 to 20	542	(18%)
	21 to 35	428	(14%)

Stroke syndrome	Other	2703	(89%)
	Lacunar	332	(11%)

Delay in randomisation	0-3 hours	849	(28%)
	> 3 hours	2186	(72%)

Delay (more detail)	0-3 hours	849	(28%)
	3-4.5 hours	1178	(39%)
	4.5-6 hours	1007	(33%)
	> 6 hours^3^	1	(0%)

Antiplatelet agents < = 48 hours before randomisation	Yes	1565	(52%)
	No	1470	(48%)

1. From January 2006, the randomisation procedure has been stratified by world region and minimises on prognostic factors within world region, using a random element.

2. For 244 patients in initial phase of the trial, NIHSS was not measured directly, but it was subsequently approximated using an algorithm (derived from subsequent patients in the trial based on clinical characteristics that were measured at baseline).

3. Protocol violation

## Baseline characteristics of patients

**Figure 1:**
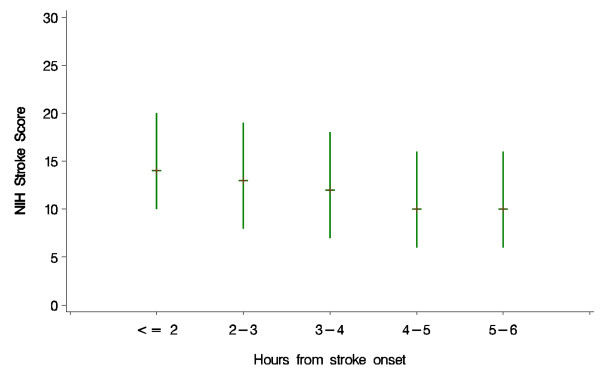
**Baseline stroke severity as assessed by NIHSS among patients randomised at different times after stroke onset**. Plot displays median and interquartile range. p value for linear regression on exact time since onset p < 0.0001.

**Figure 2:**
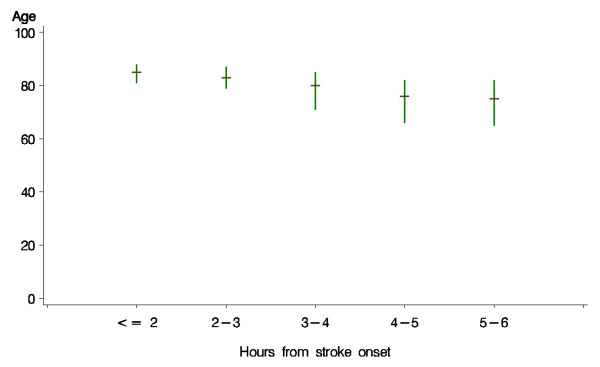
**Age among patients randomised at different times after stroke onset**. Plot displays median and interquartile range. p value for linear regression on exact time since onset p < 0.0001.

The characteristics of the patients recorded at baseline are shown, for both treatment groups combined, in Tables 2a and 3 (2b). Patients recruited within 1-2 hours of onset were statistically significantly (p < 0.0001) more likely to have a more severe neurological deficit than those recruited at later time points after onset (Figure 1). Similarly, patients recruited at earlier time points were statistically significantly older than those recruited later (p < 0.0001) (Figure 2). Additionally, the proportion of patients with a visible ischaemic lesion on the baseline imaging rose with time (data not shown).

**Table 2b:** Clinical characteristics at randomisation not used in minimisation algorithm, and recorded at randomisation

		**No**.	(%)
Cardiac rhythm	Sinus rhythm, not AF	2121	(70%)
	Atrial fibrillation	914	(30%)

Systolic BP at randomisation	< = 140 mmHg	897	(30%)
	141 to 180 mmHg	1738	(57%)
	> 180 mmHg	400	(13%)
	
	median (interquartile range)	155 (140, 170)	

Diastolic BP at randomisation	< = 80 mmHg	1537	(51%)
	81 to 110 mmHg	1420	(47%)
	> 110 mmHg	78	(3%)
	
	median (interquartile range)	80 (71, 91)	

Blood glucose^1^	< 3.5 mmol/l	7	(0%)
	3.5-6 mmol/l	532	(18%)
	6-8 mmol/l	1654	(54%)
	> 8 mmol/l	559	(18%)
	not measured (old form)	283	(9%)
	
	median (interquartile range)	7.0 (6.0, 8.0)	

Probability of being alive and independent at 6 m^2^	< 0.30	1383	(46%)
	> = 0.30	1652	(54%)
	
	median (interquartile range)	0.35 (0.12, 0.59)	

Recruited at a centre with pre-trial experience^3^	Centres without pre-trial experience	1891	(62%)
	Centre with pre-trial experience	1144	(38%)

Doctor's opinion of scan at randomisation	Normal	1792	(59%)
	Possible evidence of recent ischaemic change	701	(23%)
	Definite evidence of recent ischaemic change	542	(18%)

1. For the first 282 patients, glucose was not recorded. After patient 282, it was collected at randomisation. Glucose therefore was not available for those 282 patients

2. Probability calculated with a validated prognostic model from baseline data [14].

3. Pre-trial experience of thrombolysis is defined as having had a protocol for open label rtPA and had treated at least 3 people in the 12 months before joining the trial; 76 (49%) centres met this criterion.

## Baseline characteristics of the patients in the context of the prevailing evidence

The baseline characteristics of the patients recruited into the study changed over the course of the study as clinician's uncertainties about which types of patients should be treated changed. These changes arose from appreciation of the accumulating evidence from randomised controlled trials and consequent changes in the European Licence and clinical guidelines. At the start of the trial in 2000, rtPA was approved for use in the USA and Canada, but was not approved for use in Europe. In 2003, the European Medicines Agency granted provisional approval for the use of rtPA for patients aged less than 80 years who could be treated within three hours (and who also met a number of other criteria). In 2008, when the ECASS-3 study reported evidence of benefit for treatment between 3-4.5 hours [7], guidelines in various European countries suggested that patients aged less than 80 years could benefit even if treated within 3-4.5 hours. We estimated the number of patients in the trial that would have met either the original 2003 approval criteria or the anticipated new 2011 criteria (modified in the light of the ECASS-3 data). If we defined 'meeting the 2003 approval' as: age < 80, delay from onset to randomisation < 3 hoyrs, NIHSS 6 - 25, SBP < = 185, DBP < = 110, 2.7 < = glucose < = 22 and no history of the combination of prior stroke and diabetes mellitus, 61 patients (2%) met the criteria, and if all the same criteria were applied and the time window was extended to 4.5 hours, 350 (12.7%) met the criteria. This clearly demonstrates that the great majority of the patients in IST-3 fall outside the European approval criteria for rtPA., and hence the study will provide valuable new randomised information in many categories of patient - as well the elderly - who are currently not eligible for treatment.

## Development of the statistical analysis plan

The statistical analysis plan is being finalised at present, without reference to the unblinded data, and will be published before the code is broken in early 2012. It will take account of external evidence on factors influencing prognosis and response to treatment, the baseline characteristics of patients entering the study and advances in statistical methodology [8,9]. The SAP will define a small number of 'primary' subgroup analyses, and a larger number of exploratory secondary analyses [8]. During the course of the trial, a number of novel approaches to the analysis of ordered functional outcome data in clinical trials of stroke treatment have been suggested to improve statistical power Statistical tests, which make use of the ordered nature of outcome data, such as ordinal logistic regression and 'shift analysis', may give more reliable results [9]. However, the technique is not universally applicable and it is important that key underlying assumptions are met for the analyses to be valid [10]. The Trial Steering Committee sought statistical advice, and because the underlying assumptions would not necessarily be met in the IST3 dataset, decided an ordinal analysis was not appropriate for the primary analysis of outcome. This decision was also consistent with all the previous trials of intravenous rtPA, none of which had adopted an ordinal approach for the primary analysis. However, an ordinal analysis has been included as one of the secondary exploratory analyses. The trends in Figures 1 &2 demonstrate that the statistical analysis plan will also need to take account of these potentially confounding effects on the assessment of treatment effects overall and in subgroups.

## Reporting of results by allocated treatment

The trial will be unblinded in early 2012 and the main trial results presented at the European Stroke Conference in Lisbon in May 2012 with the aim of publishing simultaneously in a peer-reviewed journal. The trial primary results will be presented in the context of an updated Cochrane systematic review of all the relevant randomised trials. The imaging sub-study CTP/CTA/MRP/MRA results are due in early 2013. An individual patient data meta-analysis of all trials testing intravenous rtPA, including IST-3, is also being considered.

## Discussion

With 3035 patients, IST-3 is the largest ever randomised trial of intravenous thrombolysis for the treatment of acute ischaemic stroke (the two largest previous trials each recruited exactly 800 patients [7,11]. At its outset in 2000, the trial sought to improve the external validity and precision of the estimates of the overall treatment effects (efficacy and safety) of rtPA in acute ischaemic stroke and to determine whether a wider variety of patients than previously thought might benefit from this treatment. With the approval of the treatment for specific types of patients, the trial was able to evaluate the effects of treatment in patients who did not precisely meet the existing European approval for the treatment; in other words, also sought to extend the criteria for treatment. At the start of the trial, when rtPA had not yet received approval from the European regulatory authorities, patients of all ages were recruited within 3 hours. After the provisional approval for the use of rtPA in Europe in 2003 for those patients aged less than 80 years who could be treated within three hours, participating clinicians generally opted to treat patients who met the conditions of the licence, and hence only recruited only those patients in IST3 that did not strictly meet the licence criteria. The elderly have been under-represented in trials of treatments for acute stroke [12], especially thrombolysis with rtPA, where fewer than 100 patients aged over 80 years have been included in randomised trials of this treatment [13]. IST-3 has included 1617 patients aged over 80, so will provide valuable randomised evidence on the balance of risk and benefit in this much under-researched, but increasingly important age-group [12]. IST3 has shown that pragmatic trial designs with broad and flexible entry criteria can recruit large numbers of patients, even in the face of changing and burdensome regulatory regimes; the emergence of new data from other relevant trials and rapidly changing clinical practice.

## List of abbreviations

APPT: Activated Partial Thromboplastin Time; ATLANTIS: Alteplase Thrombolysis for Acute Noninterventional Therapy in Ischemic Stroke; BASC: Blood Pressure in Acute Stroke Collaboration; BP: Blood pressure; CT: Computed Tomography; CTA: CT angiography; CTP: CT perfusion; DICOM: Digital Imaging and Communications in Medicine standard; DMC: Data Monitoring Committee; DWI: Diffusion Weighted Imaging; ECASS: European Cooperative Acute Stroke Study; EPITHET: Echoplanar Imaging Thrombolytic Evaluation Trial; HU: Hounsfield Units; ICH: International Committee for Harmonisation; ISH: International Society for Hypertension; IST-3: Third International Stroke Trial; JPEG: Joint Photographic Experts Group; MR: Magnetic Resonance; MRA: MR angiography; MRP: MR perfusion; MRC: Medical Research Council; MRI: Magnetic Resonance Imaging; NIHSS: National Institute of Health Stroke Score; NINDS: National Institute of Neurological Disorders and Stroke; PT: Prothrombin Time; OR: Odds Ratio; PROBE: Prospective randomised open blinded endpoint design; rtPA: recombinant tissue plasminogen activator; SUSAR: Suspected Unexpected Serious Adverse Reaction; TSC: Trial Steering Committee.

## Competing interests

### Peter Sandercock

Has received lecture fees (paid to the Department) and travel expenses from Boehringer Ingelheim for occasional lectures given at international conferences. He was a member of the Independent Data and Safety Monitoring Board (DSMB) of the RELY trial funded by Boehringer Ingelheim and received attendance fees and travel expenses for attending DSMB meetings (paid to the Department). He is not a member of the Speaker's Panel of any company.

### Richard Lindley

Has received payment in his role as conference Scientific Committee member and for occasional lectures from Boehringer Ingelheim. He has attended national stroke meetings organised and funded by Boehringer Ingelheim.

### Joanna Wardlaw

Reimbursement for reading CT scans for ECASS III from Boehringer Ingelheim; funding to department. Is the contact reviewer for the Cochrane systematic reviews of thrombolytic treatment for acute stroke. Is director of the Brain Research Imaging Centre for Scotland. This is located within the Department of Clinical Neurosciences at the University of Edinburgh. The Centre houses a research magnetic resonance scanner which was funded by the UK Research Councils Joint Research Equipment Initiative, supplemented by grants and donations from various other sources including Novartis, Schering, GE and BI. These commercial sources contributed to the purchase of the scanner, but not the running costs or any individual studies. Has attended meetings held by Boehringer Ingelheim during the licencing of rtPA. She attended as an unpaid independent external adviser but was refunded her travel expenses and the time away from work. Has attended and spoken at national and international stroke meetings organised and funded by Boehringer Ingelheim for which she received honoraria and travel expenses. She is not on any speaker panels or panels of experts nor does she have any paid consultancies with pharmaceutical or imaging manufacturing companies.

### Karsten Bruins Slot

Received an honorarium for a lecture from Boehringer Ingelheim and had costs for participating in scientific meetings reimbursed.

### Gord Gubitz

Received honoraria and speaker fees from: Boehringer Ingelheim, Sanofi Synthlabo Aventis, Hoffman La Roche and Novo Nordisk.

### Veronica Murray

Received an unrestricted educational grant for a meeting on thrombolysis in stroke at which IST-3 was discussed.

### Graeme J. Hankey

No disclosures relating to thrombolysis.

### Eivind Berge

Has received honoraria for lectures at meetings arranged by Boehringer Ingelheim, and reimbursement for costs for attending these meetings.

## Authors' contributions

PS, RIL, JW, MD, KI designed the study and wrote the protocol, KI is the study coordinator, GC is the study statistician who prepared the analyses for this paper, DP, VS, DB and JW designed and built the computer software systems for image reading and trial data and image management. PS, RIL, MD, WW, GV, AC, AK, EB, KBS, VM, AP, GH, KM, MB, SR, GG, SP, AA, MC, PL, IK, EL recruited patients to the study, GV, AC, AK, EB, KBS, VM, AP, GH, KM, MB, SR, GG, SP, AA, MC, PL acted as National Coordinators, TC performed all the blinded follow-ups in Italy, WW assisted with follow-up of selected cases in the UK. PS drafted the manuscript and all authors commented on drafts and approved the final version

## Supplementary Material

Additional file 1 IST3 Protocol version 1_93 30th March 09_7 signed copy .pdf The current version of the trial protocol Click here for file

Additional file 2 **IST3 collabs list 141011**. List of all collaborating centres and investigators with the number of patients recruited by each centre. Click here for file
