# Supplementary material for: Update on the third international stroke trial (IST-3) of thrombolysis for acute ischaemic stroke and baseline features of the 3035 patients recruited
Source: Trials. 2011 Nov 30;12:252. doi: 10.1186/1745-6215-12-252 (PMC3286387; doi:10.1186/1745-6215-12-252)
Supplement: Additional file 1 — IST3 Protocol version 1_93 30th March 09_7 signed copy .pdf The current version of the trial protocol [file 1745-6215-12-252-S1.pdf]

Department of Clinical Neurosciences

IST-3 Study Protocol

Third International Stroke Trial: Thrombolysis for acute ischaemic stroke

**Co-Sponsors**	**University of Edinburgh & Lothian Health board NHS Trust**
**Funder**	**Medical Research Council**
**Funding Reference Number**	**G0400069**
**Chief Investigator**	**Professor Peter A. G. Sandercock, University of Edinburgh, Department of Clinical Neurosciences, Bramwell Dott Building, Western General Hospital, Crewe Road, Edinburgh, EH4 2XU**
**EudraCT Number**	**2004-000238-36**
**CTA Number**	**23105/0001/001**
**MREC Number**	**99/0/078**
**ISRCTN Number**	**ISRCTN25765518**
**Version Number and Date**	**1.93 30th March 2009**
**Details of previous amendments**	**1. Protocol October 2002, Amendments approved 20th February 2003.<br>2. Protocol 1.91 27th October 2003, Amendments approved 4th November 2003<br>3. Protocol 1.92 1st September 2005, amendments approved 13th September 2005<br>4. Protocol 1.92 1st September 2005, Amendments approved 17th November 2006.**

CONTENTS

CONTENTS 2
PROTOCOL APPROVAL 4
LIST OF ABBREVIATIONS 5
SUMMARY 6
1. INTRODUCTION 7
1.1 TRIAL HYPOTHESIS 7
1.2 BACKGROUND 7
2. STUDY DESIGN 11
2.1 Approval to start 11
2.2 Trial centre requirements 11
3. ELIGIBILITY 12
3.1 Inclusion criteria 12
3.2 Exclusion criteria 12
3.3 High blood pressure (BP) before randomisation 12
3.4 Uncertainty principle (absence of proof) 12
3.5 Consent 13
4. BRAIN IMAGING 13
4.1 Advanced imaging substudies 14
5. RANDOMISATION 14
6. TRIAL INFUSIONS 14
7. MANAGEMENT PROTOCOLS 14
7.1 Blood pressure: monitoring and intervention 14
7.2 Symptomatic intracranial bleeding 15
7.3 Asymptomatic intracranial bleeding 15
7.4 Extra-cranial bleeding 15
7.5 Allergic or hypersensitivity reactions 15
7.6 Other aspects of treatment 16
7.7 Long-term antiplatelet drugs: 16
7.8 Other treatments in hospital: 16
8. FOLLOW-UP 16
9. OUTCOME EVENTS 17
9.1 Primary 17
9.2 Secondary 17
10. ANALYSES 18
10.1 Primary analysis 18
10.2 Pre-planned subgroup analyses 18
10.3 Collaboration with systematic reviews of thrombolysis 19
11. SAMPLE SIZE 19
12. DATA AND SAFETY MONITORING 19
12.1 Definitions of Adverse Events 20
12.2 Recording and Reporting of SAEs 21
12.3 Reporting of SAEs/SARs/SUSARs 21
12.4 Breach of GCP or Trial Protocol 22
12.5 Non-serious breaches 22
12.6 Interim analyses: role of the Data Monitoring Committee 22
12.7 Compliance with Good Clinical Practice Guidelines 22
12.8 Screening Logs 23
13. ASSESSMENT AND STORAGE OF BRAIN IMAGES 23
13.1 Collection and storage 23
13.2 Assessment 23
14. ROLES AND RESPONSIBILITIES 23
14.1 Role of the Steering Committee 23
14.2 Role of the International Advisory Board 23
14.3 Role of the Management Group 24
14.4 Responsibilities of the National Co-ordinators 24
14.5 Responsibility of Principal Investigators 24
14.6 Role of the independent events adjudicator 24
14.7 Role of the image reading advisory group 24
15. INSURANCE & INDEMNITY 24

16. REPORTING, PUBLICATIONS AND NOTIFICATION OF RESULTS 25
17. NON-TRIAL THROMBOLYSIS 25
18. TRIAL ORGANISATION 25
18.1 Co-ordinating centre (for all information and queries) 25
18.2 Steering committee 26
18.3 IST-3 management group 26
18.4 International Advisory Board 26
18.5 Independent Data Monitoring Committee (DMC) 26
18.6 Independent events adjudicator 26
18.7 CT/MR image reading panel 26
19. SPONSORSHIP 26
20. REFERENCES 27
APPENDIX 1: KEY CONTACTS 30

PROTOCOL APPROVAL

Third International Stroke Trial: Thrombolysis for acute ischaemic stroke (IST-3)

EudraCT number 2004-000238-36

Signatures

Professor P. A. G. Sandercock<br>Chief Investigator	Signature	18/5/09<br>Date
Professor R. Lindley<br>Co-Chief Investigator	Signature	25/5/09<br>Date
Professor J. Wardlaw<br>Professor of Radiology	Signature	18/5/09<br>Date

LIST OF ABBREVIATIONS

APPT	Activated Partial Thromboplastin Time
ATLANTIS	Alteplase Thrombolysis for Acute Noninterventional Therapy in Ischemic Stroke
BASC	Blood Pressure in Acute Stroke Collaboration
BP	Blood Pressure
CT	Computed Tomography
DICOM	Digital Imaging and Communications in Medicine Standard
DMC	Data Monitoring Committee
DWI	Diffusion Weighted Imaging
ECASS	European Cooperative Acute Stroke Study
EPITHET	Echoplanar Imaging Thrombolytic Evaluation Trial
EU	European Union
GCP	Good Clinical Practice
HU	Hounsfield Units
ICH	International Committee for Harmonisation
IMP	Investigational Medicinal Product
ISF	Investigator Site File
ISH	International Society for Hypertension
IST-3	Third International Stroke Trial
JPEG	Joint Photographic Experts Group
MHRA	Medicines Healthcare and Products Regulatory Agency
MR	Magnetic Resonance
MRC	Medical Research Council
MRI	Magnetic Resonance Imaging
NIMP	Non-Investigational Medicinal Product
NINDS	National Institute of Neurological Disorders and Stroke
PT	Prothrombin Time
OR	Odds Ratio
PROBE	Prospective Randomised Open Blinded Endpoint Design
rt-PA	Recombinant Tissue Plasminogen Activator
SAE	Serious Adverse Event
SAR	Serious Adverse Reaction
SUSAR	Suspected Unexpected Serious Adverse Reaction
TMF	Trial Master File
TSC	Trial Steering Committee

SUMMARY

Third International Stroke Trial Protocol Summary
IST-3: A LARGE-SCALE, RANDOMISED CONTROLLED TRIAL OF THROMBOLYSIS FOR ACUTE ISCHAEMIC STROKE

Background: Data from over 5,000 patients are now available from trials of thrombolysis for patients with acute ischaemic stroke. A systematic review of these trials suggests that thrombolysis is very promising for patients who can be treated within 6 hours of stroke onset, but the balance of risk and benefit remains unclear. The majority of data are from trials of recombinant tissue plasminogen activator (rt-PA), also known as alteplase, which is licensed in Europe for use in selected patients aged under 80 years who can be treated within 3 hours. A further study, including up to 6,000 patients, is required to:

- Establish the balance of risk and benefit more precisely for thrombolytic therapy with rt-PA, especially among patients who do not exactly meet the current license criteria.

- Assess which categories of patients are most likely to benefit (by investigating: the interaction between stroke severity, early brain imaging appearances and other clinical features; and, to obtain better estimates of the duration of the 'therapeutic time window').

Trial design: International, multi-centre, Prospective, Randomised, Open, Blinded Endpoints (PROBE) design study of i.v. rt-PA vs control. Patients are entered into the trial by means of a secure web-based interface or a telephone call to a 24-hour central computerised randomisation service (Neurosciences Trials Unit, Edinburgh) with on-line minimisation to balance key prognostic factors. Repeat brain imaging will be carried out 24-48 hours following treatment to identify early asymptomatic intracranial haemorrhage. Outcome data are collected at 7 days and six months (18 month follow-up data are collected in some countries). The primary outcome is the proportion of patients alive and independent at six months (Modified Rankin 0,1 or 2). All the scans are subject to detailed central blinded review. The study is conducted in accordance with the principles of GCP and the EU directive on Clinical Trials. Details of trial procedures can be found in the trial protocol, and on the trial website (www.ist3.com).

Ethical approval: The study has received approval from the UK Multicentre Research Ethics Committee and by the local ethics committees in over 80 centres worldwide.

Funding: The trial is supported by grants from: the UK Medical Research Council, the Health Foundation (a UK medical research charity), the Stroke Association of the United Kingdom; Chest, Heart, and Stroke Scotland; University of Edinburgh; Norwegian Research Council; AFA Insurances (Sweden); The Swedish Heart Lung Fund; Karolinska Institutet; Stockholm County Council, ALF-project grants; Australian NHMRC; the Government of Poland; Swiss National Science Foundation; Swiss Heart Foundation; The Australian Heart Foundation, DesAcc; and the Dalhousie University Internal Medicine Research Fund

Sponsors: IST-3 is an independent, investigator-led trial. It is designed, and will be analysed and reported independent of the sponsors. The University of Edinburgh and the Lothian Health Board of Scotland act as joint sponsors.

1. INTRODUCTION

IST-3 is an independent, investigator-led trial. It is supported by grants from: the UK Medical Research Council, the Health Foundation (a UK medical research charity), the Stroke Association of the United Kingdom; Chest, Heart, and Stroke Scotland; University of Edinburgh; Norwegian Research Council; AFA Insurances (Sweden); The Swedish Heart Lung Fund; Karolinska Institutet; Stockholm County Council, ALF-project grants; Australian NHMRC; the Government of Poland; Swiss National Science Foundation; Swiss Heart Foundation; The Australian Heart Foundation, DesAcc; and the Dalhousie University Internal Medicine Research Fund (Canadian centre support). In the initial double-blind phase, drug and placebo for the first 300 patients were supplied by Boehringer Ingelheim. The University of Edinburgh and the Lothian Health Board act as joint sponsors. The study is designed, conducted, analysed and reported independently of the sponsors and funding agencies.

1.1 TRIAL HYPOTHESIS

That intravenous recombinant tissue plasminogen activator (rt-PA) in a dose of 0.9mg/kg (maximum 90mg) administered to patients with acute ischaemic stroke, within six hours of symptom onset, increases the proportion of people alive and independent at six months.

1.2 BACKGROUND

Acute ischaemic stroke is a major public health problem

Stroke is a common cause of death and serious disability. It has been estimated that stroke causes over four million deaths in the world each year, three million of these in developing countries, and thus is the second most common single cause of death (after ischaemic heart disease).(1) In Europe alone, a quarter of a million people will become disabled after their first stroke each year. Although deaths from cerebrovascular diseases are declining in some parts of the world, rates are increasing in others (e.g. Eastern European countries).(2) Even if age specific stroke incidence remains stable or falls slightly, as more people live into old age, the numbers of new cases of acute stroke per year may still rise.

Reducing the burden of stroke: acute treatment of stroke is unsatisfactory

Despite better treatments to prevent stroke, stroke is likely to remain a common medical emergency for the next few decades. It has been estimated that in white populations about four fifths of all strokes are ischaemic and are usually due to sudden occlusion of extra or intracranial arteries by thrombus or embolic material. Once ischaemic stroke has occurred, treatment strategies aimed at restoring the normal arterial supply are likely to have the greatest impact on reducing the burden of stroke. Current treatment, however, remains unsatisfactory. Large randomised controlled trials have demonstrated that early (within 48 hours) treatment with aspirin for acute ischaemic stroke has only modest benefit (about 1% absolute reduction in death and recurrent ischaemic stroke.(3) This treatment effect is important as aspirin is an affordable and widely practicable treatment and is probably still underused. However, more effective treatments for acute stroke are needed.

Thrombolysis for acute ischaemic stroke

Thrombolysis has been a standard treatment for acute myocardial infarction since the late 1980's. rt-PA was licensed for acute ischaemic stroke in the USA in 1996, but it was only granted a restricted licence for use in acute stroke by the European regulatory agency in 2003. Thrombolytic agents, by acting as plasminogen activators, break down the fibrin polymers of an acute thrombosis by converting plasminogen to plasmin, which in turn breaks down fibrin, releasing fibrin degradation products. The National Institute of Neurological Disorders and Stroke (NINDS) rt-PA Stroke Study group was the first trial to show evidence of benefit for thrombolysis in stroke patients.(4) Furthermore, the NINDS studies demonstrated that thrombolytic therapy for

acute stroke was only feasible after major reorganisation of the assessment of patients with suspected stroke. Subsequent trials were less promising and the extra health service requirements of an effective stroke thrombolysis service resulted in very slow uptake of treatment.(5-8) The two main barriers to widespread use of thrombolysis were the remaining uncertainty over the effect of treatment in some categories of patient and the major investment in stroke service provision required for successful and safe implementation of treatment.

Evidence on the effects of thrombolysis for patients with acute ischaemic stroke

The least biased and most precise assessment of the effects of a medical treatment is a systematic review of all the relevant randomised controlled trials. Two such reviews are available: the Cochrane systematic review(9) and the rt-PA Study Group pooled analysis (46) (Figure 2). The 2003 Cochrane systematic review included data from 8 randomised trials including 2955 patients. The rt-PA Study group pooled individual patient data on 2775 patients from 6 trials (NINDS part 1 and 2, ECASS-I; ECASS-II; and ATLANTIS Part A and B).(4-6;8;10)The Cochrane review has the advantage that it includes all trials of rt-PA in acute stroke. The rt-PA Study group pooled analysis has the advantage that it enables the effect of treatment in specific subgroups to be explored, albeit in a smaller data set. Although these two independent reviews used different methods, they both came to broadly similar conclusions which strengthen their findings.

Effect on deaths from all causes unclear

The Cochrane analyses show that rt-PA treatment was associated with a non-significant excess of deaths from all causes with rt-PA (OR 1.17, 95% CI 0.95 to 1.45 with a fixed-effect analysis). However, as there was significant heterogeneity, the overall estimate is difficult to interpret. Furthermore, the confidence intervals are wide and consistent both with a small reduction and a substantial excess of deaths. The heterogeneity may be due to many factors including time to treatment, dose of rt-PA, concomitant antithrombotic treatment and pre-treatment CT scan appearances.

Figure 1 Effect of rt-PA on the odds of death during scheduled follow-up

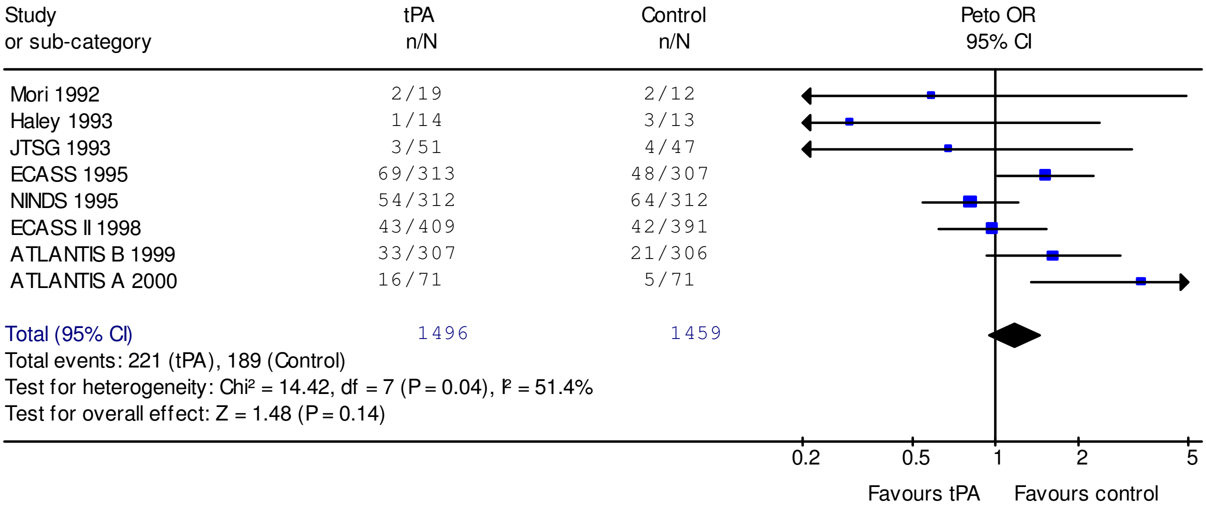

Effect of rt-PA on death or dependency

In the Cochrane review of all 8 rt-PA trials, treatment was associated with a significant reduction in the odds of being dead or dependent (OR 0.80; 95%CI 0.69 to 0.93). However, there was significant heterogeneity, and thus the estimate may not be reliable. The confidence interval was wide, and included the possibility that the benefit was very substantial or negligible. One factor that may explain some of the heterogeneity is the between-trial differences in stroke onset to treatment time. In the Cochrane review, this has been explored by analysing the results of rt-PA

for early (<3 hours) and later (3-6 hours) treatment for trials that included patients in both time periods. These analyses did not demonstrate a statistically significant difference in treatment effect between the two time periods though there was a trend for earlier treatment to be associated with better outcome (< 3 hour treatment: OR 0.69; 95% CI 0.43 to 1.09, 3-6 hour treatment: OR 0.88; 95% CI 0.73 to 1.06). The results from the individual patient meta-analysis of the rt-PA Study Group provide an opportunity to explore this effect (and other factors) in much greater detail. The rt-PA Study Group analysis investigated the association between the odds of a good outcome (based on Rankin score, Barthel Index and NIH Stroke scores) and a series of potential clinical features including such factors as onset to treatment time, age, blood pressure, stroke severity and cerebrovascular risk factors. In a multi-variate analysis the main factor associated with a more favourable outcome was earlier treatment. The odds of a favourable outcome for those treated within 90 minutes was 2.81 (95% CI 1.75 to 4.50), declining to an odds ratio of 1.15 (95% CI 0.90 to 1.47) for those treated 271-360 minutes after stroke onset (see Figure 2)

Figure 2 The adjusted odds ratio of the chance of a favourable outcome (modified Rankin score of 0-1, Barthel Index 95-100, NIHSS 0-1) at day 90 following thrombolysis with rt-PA by stroke onset to treatment time. Courtesy of the rt-PA Study group investigators (46)

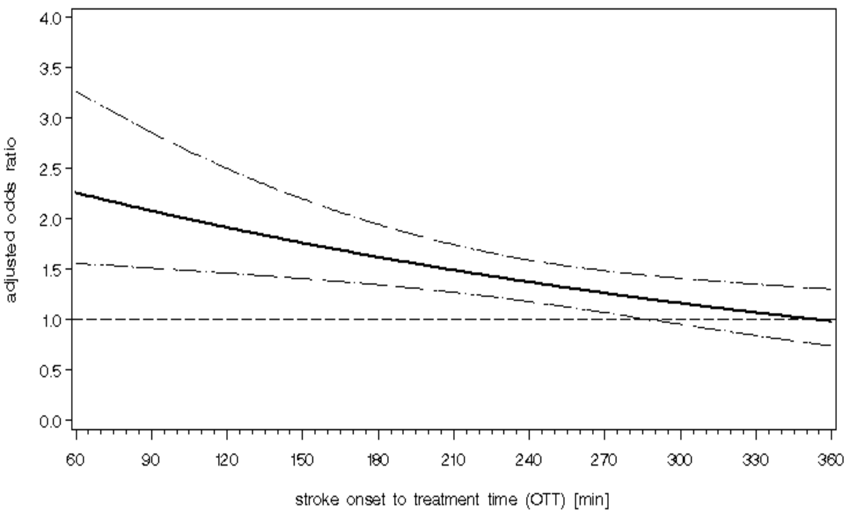

The confidence intervals about the size of the early benefit within 3 hours are wide and there is certainly scope for substantial benefit from early treatment. Similarly, the width of the confidence intervals emphasises the lack of precision and the need for further data, even under 3 hours. On the other hand, the upper confidence interval suggests that worthwhile benefit from rt-PA may extend up to six hours (for those treated between 181 and 270 minutes from stroke onset, the odds of a favourable outcome was 1.40). The rt-PA study group commented that a large randomised controlled trial with over 5,000 patients (620 < 3hrs and 4823 3-6hrs) would be required to confirm or refute these findings. Nonetheless, whether given in routine practice, or as part of a trial, these data support the notion that 'time is brain' and every effort must be made to reduce time from onset to administration of thrombolytic treatment.

Thrombolysis increases risk of symptomatic and fatal intracranial haemorrhage

In the Cochrane review, thrombolytic therapy with rt-PA was associated with a definite risk of fatal intracranial haemorrhage (OR 3.60, 95% CI 2.28 to 5.68, 2p<0.00001) with no significant heterogeneity. The rt-PA Study Group investigators assessed the effect of several clinical factors: time to treatment, age, and stroke severity on the risk of intracranial bleeding. Treatment with rt-PA was the only independent predictor. Thus, at present, there are insufficient data available to

guide clinicians on the factors that influence the occurrence of this most important side effect of treatment.

Key unanswered questions about thrombolytic therapy for ischaemic stroke
What is the 'time window' for thrombolysis? The current data suggest that the time window for treatment with thrombolysis may extend out to 6 hours from stroke onset. How long is the time window for effective treatment? Does the time window vary with patient factors? If treatment is effective up to six hours from stroke onset, a much larger number of patients would be eligible for treatment.

What is the effect of thrombolysis in older patients? Only 42 patients aged over 80 years old have been included in the rt-PA trials to date (mainly as a consequence of the 80 years age limit in the ECASS studies). About a fifth of patients with stroke are aged 80 years or older, and this under-representation of older people represents a major gap in knowledge. Although the risk/benefit ratio of rt-PA might become less favourable with increasing age because of a higher risk of adverse events, the higher risk of a poor outcome without treatment could make treatment worthwhile for some older individuals. This can only be established by the inclusion of older people in further randomised trials. IST-3 will therefore have no upper age limit.

What is the effect of thrombolysis on overall survival? The effect of rt-PA on deaths from all causes is unclear. There is a non-significant trend to an excess of deaths. Clinicians would be reassured if thrombolysis was shown to have no net detrimental effect on overall survival. If further trials confirmed that thrombolysis increased the risk of death, patients might still consider having the treatment, if those who survived the treatment had a much greater chance of being free of disability.

What predicts fatal intracranial haemorrhage? Intracranial haemorrhage is the major risk of treatment and the current trial data cannot reliably identify independent risk factors, other than choice of agent, to predict bleeding. Even the more statistically powerful individual patient meta-analysis from the rt-PA Study Group Investigators was unable to identify any clinically relevant risk factors for cerebral bleeding (other than use of the thrombolytic agent itself). Yet there is a widespread belief that clinical factors do influence risk. Many factors could influence the risk of bleeding (and hence, the potential benefits of thrombolysis) and the most important factors to explore further include: age, prior antiplatelet therapy; stroke severity; stroke subtype, whether the infarct is 'visible on CT' or not and time to treatment. Reliable data on these factors will only emerge from further randomised controlled trials, as the current systematic reviews have been unable to clarify the role of these factors.

What pre-treatment CT scan appearances predict response to treatment? Pre-treatment scans are obligatory to exclude intra-cranial haemorrhage. However, among patients with ischaemic stroke, certain features on the pre-treatment CT scan might predict the outcome of treatment. The extent and severity of any ischaemic changes on CT scanning might also provide additional prognostic information to time from stroke onset. Specific neuro-radiological features, such as the dense artery sign, might predict lack of response to treatment. Other features, such as extensive white matter change, may help identify patients at high risk of major intracranial haemorrhage with thrombolysis. Some previous analyses of pre-and post-treatment CT scans in the completed thrombolysis trials were not completely blind to treatment (and scan sequence) and the bias introduced may have over-emphasised the importance of some features.

Current clinical practice
The lack of data and clinical uncertainty about the effects of thrombolysis for acute ischaemic stroke has led to divergent expert opinion,(11-13) and as a result of this lack of consensus, the use of rt-PA is very variable.(14-16) Whilst there is strong support for the increased use of

thrombolysis from many neurologists,(17) other specialities and Emergency Medicine (EM) specialists have been more cautious(18;19) The American Emergency Physicians statement stated: 'The challenge to those who are critical of this statement is to convince the EM community as was done for MI that this should be the standard of care. It may be difficult to do this without further research.' There is also debate about the criteria for selecting patients for thrombolysis.(20) Donnan stated: 'Clearly the view [on the indication for treatment] differs from physician to physician, country to country, and continent to continent.'(11) The recent scientific statement from the American Heart Association emphasised that carefully selected patients who can be treated within 3 hours should be considered for treatment with rt-PA but 'caution should be exercised' for those with severe strokes (NIHSS > 22).(21) However, the reanalysis of the NINDS trials from the rt-PA Acute Stroke Treatment Review Panel has demonstrated that patients with severe stroke (NIHSS > 20) in the NINDS trials had an absolute benefit of about 4-5% in independent survival, which is less than in stroke of moderate severity, but is still worthwhile. A Cochrane systematic review and an NHS Health Technology Assessment both concluded that rt-PA is promising, but further large-scale controlled trials were needed before the place of this treatment in routine clinical practice could be determined.(9) (22)

The philosophy of the IST-3 collaboration is therefore that only data from large-scale randomised trials comparing rt-PA with control can dispel these uncertainties. Such uncertainty might lead to many patients being denied an effective therapy and others being treated in error. A positive and ethical approach to take, in the current environment of uncertainty and differing expert opinion, is to enrol a few thousand more patients in further randomised controlled trials. Furthermore, if IST-3 demonstrates that intravenous rt-PA can be given safely and effectively following an appropriate clinical assessment and urgent Computerised Tomographic (CT) scanning in a wide variety of emergency hospitals, treatment could be made more widely - and equitably - available to those that might benefit (and not, as at present, to the few who have access to the currently limited number of highly specialised stroke centres).

2. STUDY DESIGN

2.1 Approval to start

Hospital centres in IST-3 must have the approval of the national co-ordinator before applying for ethics approval. Appropriate local Ethics Committee approval must be sought for each participating hospital. Proof of such approval must be sent to the trial office before recruitment can be started in each centre. The trial must be run according to local procedures and law.

2.2 Trial centre requirements

A number of guidelines have stated thrombolysis should only be considered if the patient is admitted to a specialist centre with appropriate experience and expertise.(21;23) Hospitals participating in IST-3 should have an organised acute stroke service. The components of effective stroke unit care have been identified,(24) so the service should be configured along those lines and also meet local standards and guidelines. In brief, the facilities (details of these requirements are specified in the separate operations manual) should include:

- Written protocol for the acute assessment of patients with suspected acute stroke to include interventions to reduce time from onset to treatment.
- Immediate access to CT or MR brain scanning (preferably 24 hours a day).
- A treatment area where thrombolysis may be administered and the patient monitored according to trial protocol, preferably an acute stroke unit.

3. ELIGIBILITY

Patients with mild, moderate or severe strokes are potentially eligible if the following criteria are met:

3.1 Inclusion criteria

- Symptoms and signs of clinically definite acute stroke.
- Time of stroke onset is known and treatment can be started within six hours of this onset.
- CT or MRI brain scanning has reliably excluded both intracranial haemorrhage and structural brain lesions which can mimic stroke (e.g cerebral tumour).

3.2 Exclusion criteria

- The patient has previously been randomised in IST-3
- Major surgery, trauma (e.g. major fall at time of stroke) or gastrointestinal or urinary tract haemorrhage within the previous 21 days. Arterial puncture at a non-compressible site within the previous 7 days.
- Any known defect in coagulation (e.g. currently on oral Vitamin K antagonists with an INR > 1.3 **OR** other oral anticoagulant **OR** current treatment with heparin [unless APPT within normal laboratory limits] **OR** treatment with low molecular weight heparin or heparinoid).
- Known defect of clotting or platelet function (but patients on antiplatelet agents can be randomised).
- The patient is female and of childbearing potential (unless it is certain that pregnancy is not possible) or breast feeding.
- Hypo- or hyperglycaemia sufficient to account for the neurological symptoms; the patient should be excluded if their blood glucose is < 3.0 or > 20.0 mmol/L ('stick testing' is a sufficiently accurate test for this purpose).
- Symptoms considered likely to resolve **completely** within the **next few hours** (i.e. a TIA)
- Patient has had a stroke within the previous 14 days or has had treatment for acute ischaemic stroke with thrombolytic therapy within the past 14 days.
- Patient was already dependent in activities of daily living before the present acute stroke
- Patient has other life threatening illness (e.g. advanced cancer) likely to lead to death within a few months.
- Likely to be unavailable for follow-up e.g. no fixed home address.
- Patient has Blood Pressure < 90 mm Hg or > 220 mm Hg or Diastolic Blood Pressure < 40 mm Hg or > 130 mm Hg

3.3 High blood pressure (BP) before randomisation

A persistently high blood pressure can be associated with a poor outcome after stroke,(25) though high pre-treatment blood pressure was not an independent predictor of symptomatic intracranial haemorrhage with rt-PA.(26) Some patients with high blood pressure (i.e. systolic BP > 185 mm Hg and /or diastolic > 110 m Hg) can therefore be treated with rt-PA.(21) The randomisation system will only accept patients with systolic BP between 90-220 mm Hg and diastolic BP between 40-130 mm Hg. Although these data provide some guidance, the decision about whether or not to include a patient with persistently high levels of blood pressure in the trial must rest with the physicians' judgement.

3.4 Uncertainty principle (absence of proof)

Further inclusion and exclusion criteria are not specified precisely but are guided by the uncertainty principle (or absence of proof for that particular patient). If, for whatever reason, the clinician is convinced that a patient fulfilling the above criteria should be treated, the patient should be treated with rt-PA and **NOT** randomised. If the clinician is convinced that a patient should not be treated (for whatever reason), the patient should **NOT** be included in the trial.

Patients should only be randomised if they fulfil the eligibility criteria **AND** the clinician is substantially uncertain about the balance of risks and benefits of rt-PA for that individual.

3.5 Consent

IST-3 will be run according to the standards laid out in the MRC Guidelines for Good Clinical Practice in Clinical Trials (United Kingdom) and in keeping with the principles of the EU directive on Clinical Trials. These guidelines are based on the ICH Harmonised Tripartite Guideline for Good Clinical Practice and the Declaration of Helsinki. Local Ethics Committee (or local equivalent) approval is needed for each participating centre before recruitment can begin. The consent process was developed, in line with recent recommendations,(27) with consumer involvement.(28) Consent is supported by a patient (or carer) information leaflet (Appendices 2 and 3) and is adapted to local ethical requirements and the clinical state of the patient:

- If patients can understand and write, signed consent must be obtained.
- Patients who can comprehend, but are unable to write, may provide verbal witnessed consent.
- The patient's relative or spouse may act as the patient's personal legal representative and provide consent to trial inclusion, if the patient lacks the mental capacity to give fully informed consent as a result of their stroke (e.g. aphasia or decreased conscious level).
- Under certain strict criteria, if no relative is available, some local ethics committees may permit a professional legal representative, such as an independent doctor, to enable those patients unable to give consent to be recruited (this is acceptable in certain emergency situations and sometimes previously called 'a waiver of consent').(27)
- The requirements of the relevant ethics committees should be adhered to at all times.
- The signed consent form should be filed in the Investigator Site File (ISF), a copy should be filed in the patient's medical records and a copy given to the patient or carer.

4. BRAIN IMAGING

All patients MUST have a pre-randomisation brain scan to exclude intracranial haemorrhage. CT scans should cover the entire brain from the foramen magnum to the vertex with 4 – 5mm thick slices through the posterior fossa and 8 – 10mm thick for the cerebral hemispheres, with no slice gap. Scans should be windowed on a width of 80 Hounsfield Units (HU) and a centre level of 35 – 40 HU. This is particularly important if scans are to be sent as printed film. All patients (irrespective of treatment allocation) MUST have a follow-up scan at 24-48 hours. In addition a repeat scan is required if the patient deteriorates neurologically or intracranial haemorrhage is suspected for any reason. Although CT scanning is preferred, MR brain imaging is allowed provided there is sufficient radiological support in the hospital to interpret the scans and a gradient echo (T_2*) is included to exclude haemorrhage (haemorrhage can be overlooked on several other types of MR imaging sequence) and Diffusion Weighted Imaging (DWI) is required to identify the recent infarct. All scans performed during the first 7 days following randomisation are to be sent to Edinburgh for coding. The two sets of CT scans per patient (more, if the patient had extra scans due to suspected complications) are to be sent to the Edinburgh trials office, either by post, or (subject to certain conditions) by electronic transfer of DICOM files (details of methods of file transfer and copies of the Scan transfer forms are given in the trial operations manual). If sending a hard copy film, the original is to be sent, as this allows better conversion to an electronic file (a copy should be made and kept at the treating hospital). Hard copy scans will be digitised and converted to DICOM files. All images will be coded with all original identifiers stripped from the record. Each scan can then be assessed, blind to patient details, and to whether the scan is pre- or post treatment. Each scan will be assessed by an international panel of expert radiologists by means of an internet web-based computer system.

4.1 Advanced imaging substudies

IST-3 will permit advanced imaging substudies in centres with appropriate facilities and local expertise. Such studies could include CT angiography, MR diffusion and perfusion imaging, carotid duplex and transcranial doppler imaging. Any such proposed sub-studies must be approved by the IST-3 Trial Steering Committee.

5. RANDOMISATION

The clinician enters patients via a secure web based randomisation system (www.ist3.com) or by telephone call to an automated randomisation system available 24 hours a day. The randomisation system requests a few key items of baseline data, which are then entered with the telephone keypad. When the data have been entered and checked, the computer generates the treatment allocation. From the start of the trial until December 2005, the system included a standard minimisation algorithm which ensured that the treatment groups are balanced for key prognostic factors.(29) The algorithm balanced allocation on stroke severity (calculated as the patient's predicted probability of a poor outcome, calculated from a validated prognostic model based on key clinical variables measured at baseline),(30). After review of the degree of balance and severity of the strokes in patients in the trial, the randomisation algorithm was modified in January 2006. From that day onwards, treatment allocation has been stratified by world region and minimised on sex, age, NIHSS, stroke syndrome, delay to randomisation and use of antiplatelet drugs within the past 24 hours, with a random element. Patients allocated 'immediate rt-PA' should be treated as soon as possible after the randomisation call is completed.

6. TRIAL INFUSIONS

All patients should have intravenous access in place and be administered intravenous fluid therapy according to local acute care protocols. Patients allocated 'immediate rt-PA' should be given recombinant tissue-type plasminogen activator (Alteplase, Boehringer Ingelheim; or Activase, Genentech) in a total dose of 0.9mg per kg of body weight up to a maximum of 90mg. Ten per cent of the dose is given as an intravenous bolus delivered over one minute followed by the rest of the infusion over the next 60 minutes. Patients allocated 'control' must avoid treatment with rt-PA and should receive stroke care in the same clinical environment as those allocated 'immediate rt-PA'. Both treatment groups must have their blood pressure monitored closely over the first 24 hours, according to the IST-3 protocol, and this must be documented. Both groups should receive the same general supportive care.

7. MANAGEMENT PROTOCOLS

All patients entered in the trial, whether allocated rt-PA or control, must be managed according to local acute stroke care protocols, in the same clinical environment. Such protocols are not specified by the trial, but will generally include the components of effective stroke unit care.(24) Soon after admission, intravenous access, monitoring of physiological variables, correction of any abnormalities, and where clinically appropriate, intravenous fluid therapy should be initiated.

7.1 Blood pressure: monitoring and intervention

The NINDS group specified a detailed protocol for the active lowering of blood pressure, though it was unclear whether this policy was beneficial or harmful to patients in the trial.(31) The Blood Pressure in Acute Stroke Collaboration (BASC), have since reviewed all the relevant randomised controlled trials of blood pressure lowering in acute stroke(32) and concluded (as did the International Society for Hypertension [ISH])(33) that there were no data from reliable randomised controlled trials to guide the management of high blood pressure in patients with acute stroke. Blood pressure tends to fall in the acute phase of stroke and in view of the conclusions of the BASC and ISH, no particular IST-3 protocol for blood pressure management will be specified. To monitor any interaction between blood pressure and response to treatment in IST-3, data on blood pressure levels and the use of blood pressure lowering treatments will be collected. This aspect of the trial will be monitored by the Data Monitoring Committee.

7.2 Symptomatic intracranial bleeding

Intracranial haemorrhage should be suspected if any of the following occur during the infusion or within 24 hours of randomisation:

- Neurological deterioration.
- New headache.
- New acute hypertension.
- New nausea or vomiting.
- Sudden decrease in conscious level.

If any of these events occur, any rt-PA infusion should be stopped and the patient examined for possible reasons for the deterioration. Blood should be taken to measure prothrombin time (PT), activated partial thromboplastin time (APPT), fibrinogen, full blood count and group and save serum. CT scanning must be performed immediately, irrespective of the allocated treatment group. If CT scanning confirms intracranial haemorrhage, rt-PA must not be restarted. Management should follow local protocols and will usually require consultation with a haematologist and a neurosurgeon. For patients who have received rt-PA there is no reliable evidence available to recommend any one treatment strategy over another, but fibrinolytic inhibitors such as tranexamic acid may be useful. In the rare instance that fibrinogen levels are low (<1g/L) after rt-PA therapy, cryoprecipitate (containing fibrinogen and factor VIII) may be required.(34) Fibrinogen assays vary but the Clauss technique is considered the best method if available.(35)

7.3 Asymptomatic intracranial bleeding

If asymptomatic intracranial bleeding (haemorrhagic transformation of the infarct or parenchymatous haematoma) is detected on the repeat CT or MR scan performed at about 24 hours after randomisation, no specific action is needed, but it may be necessary to delay the start of long-term antiplatelet or anticoagulant therapy. The degree of delay is a matter for the responsible clinician to determine, but will be influenced by factors such as the degree and extent of haemorrhage, the patient's clinical condition, the nature of the planned treatment and the indication for its use.

7.4 Extra-cranial bleeding

If significant extra-cranial bleeding develops, any rt-PA infusion must be stopped immediately. Blood should be taken to assess prothrombin time (PT), activated partial thromboplastin time (APPT), fibrinogen, full blood count and cross match. Appropriate supportive therapy should be given, with monitoring of blood pressure, maintenance of circulating blood volume with intravenous fluids and transfusion of blood as appropriate. The results of the investigations will guide emergency treatment. Management should follow local protocols and will usually require consultation with a haematologist. For patients who have received rt-PA there is no reliable evidence available to recommend any one treatment strategy over another, but fibrinolytic inhibitors such as tranexamic acid may be useful. If fibrinogen levels are low (<1g/L) cryoprecipitate (containing fibrinogen and factor VIII) may be required.(34) Fibrinogen assays vary but the Clauss technique is considered the best method if available.(35)

7.5 Allergic or hypersensitivity reactions

Anaphylactic reactions can occur following rt-PA administration for acute ischaemic stroke, but occur rarely.(36) If there are any signs of anaphylactic response or hypersensitivity (e.g. peri-orbital swelling, tongue swelling, urticarial rash) any rt-PA infusion should be stopped immediately. Patients require urgent medical assessment ('airway, breathing and circulation'). Treatment will depend on the severity of the reaction. Intravenous steroids and antihistamines

may be sufficient for mild reactions. Adrenaline (nebulised or intramuscular) and intubation may be required for severe reactions. Local advice from the emergency medicine team should be sought. All such reactions should be recorded on the 7-day hospital form.

7.6 Other aspects of treatment

Antithrombotic treatment should not be given within the first 24 hours of the start of rt-PA treatment: There is some evidence to suggest that early aspirin, given with thrombolytic therapy, may increase the risk of death.(37) Antithrombotic treatment (antiplatelet drugs and heparin) should therefore be avoided in the first 24 hours after start of trial treatment for patients who have received rt-PA. Patients treated with rt-PA should first have a repeat CT or MR brain scan, performed at 24-48 hours after treatment, and start long-term antiplatelet therapy with aspirin or other agents, only if the second CT has excluded intracranial haemorrhage. Patients allocated control should start long-term antiplatelet therapy with aspirin (or other effective antiplatelet agent) after randomisation according to usual practice. There are no data to suggest that this delay in initiating antiplatelet drugs materially disadvantages rt-PA allocated patients. The modest benefit of aspirin, given at 24-48 hours after onset of stroke symptoms, was similar to that when given within the first few hours.(3) Conversely, the earlier use of aspirin for patients allocated control is therefore unlikely to introduce a major difference between rt-PA and control groups and will anyway reflect usual clinical practice for control patients. All antithrombotic medication used in the first week following treatment will be recorded on the 7 day trial form to monitor deviation from the protocol and assess risk factors for side effects.

7.7 Long-term antiplatelet drugs:

Unless there is a clear contraindication, all patients should be considered for long-term antiplatelet therapy with aspirin (or other effective antiplatelet) for routine secondary prevention of vascular events.(38) Treatment should not be started until 24 hours after any rt-PA infusion (see above). At discharge, all patients will be given a card recording their participation in the study and their General Practitioners should be informed by letter (see example in Appendix).

7.8 Other treatments in hospital:

All other aspects of treatment are at the discretion of the responsible clinician.

8. FOLLOW-UP

All patients will be followed up, whether they complied with their treatment or not. Follow-up will be at seven days, hospital discharge, transfer to another hospital or death, whichever occurs first (see Appendix: Hospital Follow-up Form). The Hospital Co-ordinator at each collaborating centre must complete the hospital follow-up form for each patient, and send it to the IST-3 Trial Office, enclosing a copy of the pre-and all post-randomisation CT and MRI scans.

Six months after randomisation, General Practitioners (or Hospital Co-ordinators) will be contacted by the IST-3 Trial Office staff to check that their patient is alive and that they may be approached for follow-up. If appropriate, the IST-3 Trial Office staff will then mail a postal questionnaire to patients, to record dependency and health related quality of life. The exact procedures for follow-up in each country will be decided by the National Co-ordinator and the IST-3 Management committee. Central follow-up (telephone or postal) has been found to be cost-effective, efficient and also ensures blinding of the assessment. If a patient dies after a hospital follow-up form has been completed (up to 7 days from randomisation), and within 6 months of randomisation, the clinician can conveniently inform the IST-3 Trial Office by completing and returning a simple form to reduce the risk of the co-ordinating centre mailing a questionnaire to a patient who has died (see Appendix: 6 Month Follow-up From). The precise date of death will be very important for survival analyses.

To assess the durability of any treatment benefit beyond 6 months, patients recruited in the UK (and in other countries where appropriate funding has been obtained) will be followed up one year after the six month assessment and annually thereafter (dependent on sufficient funding). These data will also permit more detailed health economic modelling and to test the hypothesis, that the level of disability at six months predicts long-term survival.

Patients may withdraw consent to participate in the trial at any stage. This may involve withdrawal from trial treatment (in which case any rt-PA infusion should be stopped) or withdrawal from trial follow-up. If the latter, it is essential to obtain their consent at the point of withdrawal to obtain follow-up information on their outcome from other sources, e.g. from their hospital records, their general practitioner or central health services data.

9. OUTCOME EVENTS

9.1 Primary

The primary measure of outcome is the proportion of patients alive and independent (i.e. Modified Rankin Score 0-2) assessed by validated postal/telephone questionnaires six months after randomisation.(39)

9.2 Secondary

a) Fatal events within 7 days

- **Deaths from any cause**
- **Deaths within 7 days, subdivided by cause of death**. Deaths attributed to neurological causes will be categorised as follows: death due to swelling of the initial infarct; death due to intracranial haemorrhage; death due to the initial stroke, but not attributable to infarct swelling or haemorrhage; death due to recurrent ischaemic stroke; or death due to recurrent stroke of unknown type.

b) Non-fatal events within 7 days. The occurrence of one of the following events within 7 days, in a patient alive at 7 days:

- **Symptomatic intracranial haemorrhage.** In a patient with ***either*** a clinically important worsening of their deficit measured on a valid stroke scale, ***or*** the occurrence of a clinical syndrome suggesting recurrent stroke***,*** together with the presence of significant intracranial haemorrhage on a CT or MR scan performed within 7 days of randomisation considered sufficient to have contributed to the deterioration.
- **Recurrent ischaemic stroke**. Further stroke in a different vascular territory to the index stroke, according to clinical features. The diagnosis must be supported by brain imaging to exclude haemorrhage (but not necessarily to confirm the vascular territory of the new infarct).
- **Recurrent stroke of unknown type**. Further stroke in a different vascular territory to the index stroke, according to clinical features, but with no brain imaging or autopsy performed.
- **Neurological deterioration attributed to swelling of the initial ischaemic stroke**. In a patient with relevant clinical deterioration, the presence of significant cerebral oedema (i.e complete ventricular effacement, midline shift or obliteration of the basal cisterns) on a post-randomisation CT scan (or MR) performed within 7 days of randomisation.
- **Neurological deterioration not attributable to swelling of the initial ischaemic stroke or haemorrhage**. A patient with relevant clinical deterioration, but no evidence on CT or MR of significant swelling or haemorrhage.

c) Other events within 7 days

- **Major extracranial haemorrhage** (i.e. fatal, severe enough to require transfusion or operation, or an absolute decrease in haemoglobin $\geq$ 5 g/dL or a decrease in haematocrit of $\geq$ 15% or bleeding associated with persistent or serious disability).

- **Asymptomatic intracranial haemorrhage identified by routine repeat brain imaging.** The presence of any intracranial haemorrhage on a CT scan (or MR scan) performed within 7 days of randomisation with no clinical deterioration (i.e. no corresponding worsening of neurological deficit, and no evidence of recurrent stroke)

d) Outcome at six months (and, for UK patients, at 18 months and annually thereafter)

- Number of patients dead from any cause within six months
- Number of patients dead from a vascular cause (includes death due to bleeding) within six months
- Number of patients making a complete recovery from the stroke (defined by simple recovery question)(40)
- Oxford Handicap Score (Modified Rankin)(39)
- 'Dependency' question.(40)
- EQ-5D (EuroQol) questionnaire.(41;42)
- Residence at six months (i.e. at home, still in hospital, long-term geriatric ward, nursing home, residential home, with relatives or other)

10. ANALYSES

'Intention-to-treat' analyses will be used throughout.

10.1 Primary analysis

The primary analysis will be a comparison of the proportion of patients in each group who are alive and independent at six months (Modified Rankin 0,1 and 2), for all those allocated rt-PA versus all those allocated control.

10.2 Pre-planned subgroup analyses

Analyses will be performed of the effect of treatment at six months among all those allocated rt-PA versus all those allocated control, subdivided by the following baseline features:

- Time from onset to randomisation (0-3 vs. 3-6 hours)
- Age
- Gender
- Clinical stroke syndrome using the OCSP classification(43)
- Presence or absence of atrial fibrillation (AF)
- Pre-randomisation brain imaging appearances (extent of visible infarction, visible infarct versus not, small vessel disease and atrophy)
    a) as assessed by the randomising clinician (recorded at baseline), and
    b) as assessed by independent blinded review of the pre-randomisation scan
- Use of antiplatelet drug treatment at the time of randomisation
- Stroke severity according to the NIHSS.
- Blood pressure at randomisation
- Randomisation in a centre with prior experience (treatment of more than 3 patients with thrombolysis in the 12 months prior to the start of the trial) versus not
- Randomisation during the double blind start-up phase vs. randomisation during the main (open) phase.

A variety of other subsidiary analyses, will be performed of the effect of treatment on: death from any cause within the first seven days; death from any cause at six months; death from vascular causes at six months; fatal intracranial haemorrhage; complete recovery at six months; outcome as measured by the Oxford Handicap Score.(39) Treatment effects on the primary outcome and

secondary outcomes, subdivided by other baseline features will be performed as appropriate, with due allowance for their exploratory nature.

10.3 Collaboration with systematic reviews of thrombolysis

When the main report of IST-3 has been published, the IST-3 group will collaborate with the authors of the Cochrane systematic review and with the rt-PA Study Group to include the trial data from the trial in their analyses.

11. SAMPLE SIZE

The IST-3 has a planned sample size of up to 6,000 patients. Assuming a power of 80%, an alpha level of 5%, and the same control event rate as observed in the trial to September 2005 (confidential data), with 6000 patients, mostly treated between 3 & 6 hours of onset, the trial could detect a 3% absolute difference in the primary outcome (the proportion of patients dead or dependent at 6 months). This absolute difference is clinically worthwhile, is consistent with the effect size observed among patients randomised between 3 & 6 hours of stroke onset in the Cochrane review of the rt-PA trials. It is also comparable with the absolute benefit seen with thrombolytic therapy for acute MI. If 3500 patients were recruited, the trial could detect a 4% absolute difference in the primary outcome. A sample size of 1000 patients could detect a 7% absolute difference in the primary outcome, which is consistent with the effect size among patients randomised within 3 hours of stroke in the Cochrane review.

Amendment in the light of recruitment by 2007: The trial began recruitment with the feasibility phase in 2000. The main trial began in 2005 with recruitment scheduled to end in 2009. In 2007, it became clear that recruitment of 6000 patients by 2009 was not realistic. The Medical Research Council therefore awarded an extension to funding to permit recruitment to continue until mid 2011. with a revised recruitment target of 3100 which would yield 80% power to detect an absolute difference of 4.7% in the primary outcome.

12. DATA AND SAFETY MONITORING

The Principal Investigator is responsible for the detection and documentation of events which meet the criteria and definitions as detailed below.

Full details of contraindications and side effects that have been reported following administration of the trial drug can be found in the Summary of Product Characteristics (SoPC). Details of the contraindications and side effects are given in the table below:

**Known Adverse Events following treatment with Actilyse**	
**Event**	**History**
Bleeding	Frequently<br>superficial bleeding, normally from punctures or damaged blood vessels, Ecchymosis, epistaxis and gingival bleeding<br><br>Occasionally<br>internal bleeding into the gastro-intestinal or uro-genital tract, retro-peritoneum or CNS or bleeding of parenchymatous organs.
Symptomatic intracerebral haemorrhage	Up to 10% of patients
Embolisation or thrombotic embolisation which may	Rare

lead to corresponding consequences (e.g. renal failure in the case of renal involvement).	
Arrhythmias	In patients receiving Actilyse for MI
Cardiac failure<br>Recurrent ischaemia<br>Angina<br>Cardiac arrest<br>Cardiogenic shock<br>Reinfarction<br>Valve disorders (e.g. aortic valve rupture)<br>Pulmonary embolism	Following MI or Pulmonary Embolism
Nausea<br>Vomiting<br>Drop in blood pressure<br>Increased temperature	These reactions can also occur as concomitant symptoms of myocardial infarction
Events related to the central nervous system (e.g. convulsions)	Reported in isolated cases, often in association with concurrent ischaemic or haemorrhagic cerebrovascular events.
Anaphylactoid reactions - rash, urticaria, bronchospasm, angio-oedema, hypotension, shock or any other symptom associated with allergic reactions	Rare
Transient antibody formation to Actilyse	Observed in rare cases and with low titres, but a clinical relevance of this finding could not be established.

12.1 Definitions of Adverse Events

Serious adverse event (SAE) - is any untoward medical occurrence in a subject to whom a medicinal product has been administrated. This includes occurrences which are not necessarily caused by or related to that product.

Serious adverse reaction (SAR) - any untoward and unintended response, in a subject to an investigational medicinal product, related to any dose administered to that subject. This is known as an adverse drug reaction. Adverse reactions to a particular drug may be already listed in the Investigator Brochure for the drug or the SPC.

Suspected unexpected serious adverse reaction (SUSAR), where the nature and severity of the reaction is not consistent with the information about the medicinal product/medical device set out in the summary of product characteristics for that product

Definition of serious: An adverse reaction or adverse event is serious (termed SAE, SAR or SUSAR) if it:

- Results in death

- Is life threatening (subject was at risk of death at the time of event)
- Requires hospitalisation or prolongation of existing hospitalisation
- Results in persistent or significant disability or incapacity
- Consists of a congenital anomaly or birth defect.

It is the principal investigator's responsibility to decide whether the event was related (resulted from administration of any of the research procedures) or unexpected (type of event not listed in the protocol as an expected occurrence).

12.2 Recording and Reporting of SAEs

Due to the serious nature of ischaemic stroke and the many associated serious adverse events that arise following an ischaemic stroke and the many known serious adverse reactions associated with administration of the trial drug, in IST-3 there is therefore no requirement for SAE's and SARs to be expeditedly reported to the sponsor within 24 hours. However, relevant SAE's should be reported on the 7 day Follow-up Form. All relevant SAE's occurring within 7 days of randomisation are adjudicated by the events adjudication committee.

12.3 Reporting of SAEs/SARs/SUSARs

Relevant SAE's should be recorded on the 7 day Follow-up form. If the Investigator has made an assessment and suspects that that the SAE/SAR is unexpected, the Investigator must report the information to the Sponsor within 24 hours. **The procedure on how to report unexpected SAEs/SARs to the Sponsor is available in the trial manual.**

SARs that are unexpected (SUSAR) will be reported by the Sponsor to the main REC, MHRA and the Data Monitoring Committee. SUSARs that are fatal or life threatening will be reported within 7 days and all other SUSARs will be reported within 15 days. All Principal Investigators will be informed of all SUSARs reported to the Sponsor.

Expedited reports of other events

The European Commission guidance recommends that the following safety issues are also reported to the sponsor in an expedited fashion:

1. An increase in the rate of occurrence or a qualitative change of expected SAR, which is judged to be clinically important.
2. Post-study SUSARs that occur after the trial subject has completed a clinical trial and are notified by the Investigator to the sponsor.
3. New events related to the trial or the development of the IMPs and likely to affect the safety of the trial subjects.
4. Recommendations of the Data Monitoring Committee (DMC) where relevant for the safety of the trial subjects.
5. Any reaction due to a Non Investigational Medicinal Product (NIMP) that is likely to affect the safety of the trial subjects.

If identified, the Investigator must report these safety issues to the sponsor in the same way as described in section 12.4. The sponsor is responsible for informing the MHRA and the main REC of these safety issues within the same timelines as described in section 12.4.

12.4 Breach of GCP or Trial Protocol

It is the responsibility of the Chief Investigator (CI), Principal Investigator(s) (PI), the study team and the sponsor continually to monitor the conduct of the trial. See ACCORD Standard Operating Procedure for escalation and notification of serious breaches of GCP or the trial protocol (SOP25)

In accordance with the Clinical Trial Regulations 2004, serious breaches of GCP or the trial protocol must be reported to the Medicines and Healthcare Products Regulatory Agency (MHRA). Serious Breaches are defined as a breach which is likely to significantly affect:

- The safety or physical or mental integrity of trial subjects; or
- The scientific value of the trial

Any potential breaches of GCP or the protocol identified must be reported to the sponsor within 24 hours in accordance with the sponsor protocol and should include the following information

- The country ID, site ID and name of the PI
- The patient details (if applicable)
- An explanation of how the breach was identified
- Details of the breach
- Details of any corrective action
- An assessment of the impact to participant safety and/or scientific integrity of the trial.

If the breach is assessed as 'serious' it will reported to the MHRA within 7 days and resolved in accordance with the regulations.

12.5 Non-serious breaches

Where the sponsor does not assess the breach as serious i.e. a deviation from the protocol that does not result in harm to the trial subjects or significantly affect the scientific value of the trial, the case should be documented in the 7 Day form for the trial and the Trial Master File. Appropriate corrective and preventative actions should also be taken.

12.6 Interim analyses: role of the Data Monitoring Committee

During the period of recruitment into the study, interim analyses of the proportion of patients alive and independent and the numbers of total deaths at six months and analyses of other major outcome events will be supplied, in strict confidence, to the chairman of the data monitoring committee, along with any other analyses that the committee may request. In the light of these analyses, the data monitoring committee will advise the chairman of the steering committee if, in their view, the randomised comparisons have provided both (i) 'proof beyond reasonable doubt' that for all, or some, the treatment is clearly indicated or clearly contra-indicated and (ii) evidence that might reasonably be expected to materially influence future patient management. Appropriate criteria of proof beyond reasonable doubt cannot be specified precisely, but the DMC will work on the principle that a difference of at least 3 standard errors in an interim analysis of a major outcome event (e.g. death from all causes or independent survival at six months) may be needed to justify halting, or modifying, a study before the planned completed recruitment. This criterion has the practical advantage that the exact number of interim analyses would be of little importance, and so no fixed schedule is proposed.(44) Following a report from the DMC, the steering committee will decide whether to modify entry to the study (or seek extra data). Unless this happens however, the steering committee, the collaborators and central administrative staff will remain ignorant of the interim results.

12.7 Compliance with Good Clinical Practice Guidelines

The trial will conform to the MRC Guidelines for Good Clinical Practice in Clinical Trials.(45) Trial data will be checked for validity and internal consistency and various measures will be taken to identify any scientific misconduct (details of such measures remain confidential for obvious reasons).

In addition, centre audit visits may be carried out and primary records may be inspected as necessary.

12.8 Screening Logs

Screening logs are not part of the IST-3 data collection process. In IST-3, it is likely that only a small proportion of the very large numbers of people presenting with symptoms of suspected acute stroke will prove to be eligible and an even smaller proportion are likely to volunteer to enter the study. Not collecting screening log data will not introduce bias into the assessment of the effects of treatment. Furthermore, the effort of collecting the screening log data may represent a substantial effort for participating centres. This effort will not, in this particular trial, serve to improve the quality of the data collected on those patients that are recruited to the trial and may divert limited time away from the pressing tasks of treating patients or recruiting patients to the study. In the view of the chief investigators, there is therefore no justification to collect screening log data.

13. ASSESSMENT AND STORAGE OF BRAIN IMAGES

13.1 Collection and storage

CT and MR brain scans at baseline and follow-up are to be sent by secure mail or electronic means to Edinburgh. Anonymised digital copies of these scans will be stored on computer servers for analysis and archiving. The systems have been designed to ensure the highest levels of data security and patient confidentiality, and will be further enhanced if future technological advances permit it. The enhancements to the current system may include the use of e-Science and Grid technologies if they prove to be superior to current systems. The use of e-Science infrastructure within the MRC Neurogrid project for the IST-3 imaging data could: ensure more reliable, secure and confidential archiving of the imaging data; connect sites for rapid and secure flow of data; enable distributed data analysis with image analysis tools; enhance collaborative working between members of the research team; and, improve the power and applicability of studies. Centres who are routinely performing MR diffusion with perfusion (with or without MR angiography) or CT with perfusion (with or without angiography) are requested to also provide the perfusion and/or angiographic data via the same image data transfer route along with the plain CT or plain MR data.

13.2 Assessment

All brain scans (baseline and follow-up) are to be assessed by at least one expert reader, by means of a web-based image assessment tool which presents anonymised images to the reader. The image data remain on the trial server; the system presents anonymous images in Joint Photographic Experts Group (JPEG) format (with no personal or demographic or other information) together with a structured questionnaire on the same screen to the assessor. The assessor then records their interpretation of the scan by means of the on-screen questionnaire. The scan interpretation is then stored directly on the secure IST3 trial database, with no need for email, fax or postal transmission of data. The Image Reading Advisory panel will advise on the conduct of this work, on the size of the CT reading panel, and on the selection of readers.

14. ROLES AND RESPONSIBILITIES

14.1 Role of the Steering Committee

The Committee will be responsible for overseeing the conduct of the trial. It shall be constituted and operate as laid out in the MRC Guidelines for Good Clinical Practice.(45)

14.2 Role of the International Advisory Board

This will constitute the National Co-ordinators from each participating country, representatives of other major trials and other individuals with relevant expertise who may be co-opted as appropriate. The Board will be chaired by Chairman of the Trial Management Group. The Board fulfils two roles:

a) to advise the trial management team and the trial steering committee on matters relevant to the trial, and b) to enable appropriate representation of the National Co-ordinators views on the trial. Advice from the Board to the steering committee and management committee is not binding.

14.3 Role of the Management Group

The group is responsible for all aspects of day to day management of the trial and is based at the Neurosciences Trials Unit at Edinburgh University. It is responsible for: the recruitment of trial centres; provision of training material for the collaborating centres; organising trial meetings and training meetings; provision of trial materials; data collection, checking and data entering; trial analysis; co-ordinating the production of trial reports and publications.

14.4 Responsibilities of the National Co-ordinators

National Co-ordinators will represent the IST-3 in their country. The National co-ordinator should: approve new local co-ordinators before they submit any local ethics committee application; undertake the centralised follow-up at six months in their country; liaise with the IST-3 Management Group over the conduct of national follow-up procedures; help maintain a high profile for IST-3 in their country and encourage appropriate recruitment; attend meetings of the International Advisory Board to represent the views of participants in their country.

14.5 Responsibility of Principal Investigators

Principal Investigators will represent the IST-3 in their centre (hospital). It is expected that Principal Investigators work in a well organised stroke service, preferably including a stroke unit. The Principal Investigators should: liaise with the National Co-ordinator prior to any local ethics application and trial start-up in their hospital; maintain a high standard of stroke assessment and 'fast-tracking' of potential participants in their hospital, supported by a written protocol; liaise with local neuroradiology or radiology colleagues to ensure immediate access to CT brain imaging; liaise with appropriate emergency medicine colleagues; be responsible for continuous medical education to maintain appropriate high standards of care for patients considered and randomised in the trial (this will usually involve regular meetings with medical, nursing, allied health care staff in the emergency department and stroke unit); ensure compliance with Good Clinical Practice Guidelines.

14.6 Role of the independent events adjudicator

An expert independent clinician will review - blinded to treatment allocation - clinical and radiological information on any significant cerebral event that occurs up to 7 days after randomisation. The classification of such events will be compared with that assigned by two senior members of the trial management staff and any differences will be resolved by discussion. The agreed assessment of each event will form part of the data reviewed in confidence by the Data Monitoring Committee.

14.7 Role of the image reading advisory group

All brain scans (baseline and follow-up) will be reviewed by a panel of expert readers. The methods to be used by the panel of readers, the overall conduct of the image reading process and the interpretation of the findings at the end of the study will be guided by the image reading advisory group. Suitably qualified experts will be invited to join the group which is chaired by Professor Wardlaw.

15. INSURANCE & INDEMNITY

15.1 Sponsorship

The Study is sponsored by the University of Edinburgh ("the University") and Lothian Health Board NHS Trust ("LHB") (together the "Sponsors").

The Sponsors are responsible for ensuring proper provision has been made for insurance or indemnity to cover their liability and the liability of the chief investigator. The following arrangements are in place to fulfil the Sponsors' responsibilities.

15.2 Negligent Harm caused by the Study Design

The Study has been designed by researchers employed by the University of Edinburgh, and the University has insurance in place (which includes no-fault compensation) for negligent harm caused by the Study design.

15.3 Clinical Negligence

Individual Centres involved in the Study will be liable for clinical negligence and other negligent harm to individuals taking part in the Study and covered by the duty of care owed to them by the Centres concerned. The Sponsors require individual Centres participating in the Study to arrange for their own insurance or indemnity in respect of these liabilities. **UK Centres:** Centres which are part of the United Kingdom's National Health Service will have the benefit of NHS Indemnity. **Non UK Centres:** Centres outside the United Kingdom will be responsible for arranging their own indemnity or insurance for their participation in the Study, as well as for compliance with local law applicable to their participation in the Study.

15.4 UK Centres

Centres which are part of the United kingdom's National Health Service will have the benefit of NHS indemnity.

15.5 Non UK Centres

Centres outwith the United Kingdom will be responsible for arranging their own indemnity or insurance for their participation in the study, as well as for compliance with local law applicable to their participation in the Study.

16. REPORTING, PUBLICATIONS AND NOTIFICATION OF RESULTS

Publication in the names of all collaborators.

The success of this study depends entirely on the collaboration of a large number of doctors, nurse and patients. For this reason the credit for the main results will be given, not to the central trial co-ordinators, but to all wholehearted collaborators in the study. The primary trial publication will be drafted by a writing committee whose membership has been approved by the steering committee. The manuscript must be approved by the steering committee before submission for publication.

17. NON-TRIAL THROMBOLYSIS

The IST-3 Group recommends, where required by local protocol, that any non-trial thrombolysis treatment for stroke is registered, in relevant post-marketing studies such as SITS-MOST (http://www.acutestroke.org).

18. TRIAL ORGANISATION

18.1 Co-ordinating centre (for all information and queries)

IST-3 Co-ordinating Centre, Department of Clinical Neurosciences, University of Edinburgh, Bramwell Dott Building, Western General Hospital, Edinburgh, UK, EH4 2XU. email: ist3@skull.dcn.ed.ac.uk, telephone: +44 (0)131 537 2793 fax: + 44 (0)131 332 5150; web-site http://www.ist3.com.

18.2 Steering committee

Independent Chairman: Professor Colin Baigent, University of Oxford; Independent members: Dr Pippa Tyrrell (Manchester University), Professor Gordon Lowe (Glasgow University); Co-principal Investigators: Professor Peter Sandercock (Edinburgh University); Professor Richard Lindley (Professor of Geriatric Medicine, University of Sydney); Statistician: Dr Stephanie Lewis; Lead Neuroradiology Investigator: Professor Joanna Wardlaw (Edinburgh University); Trial Manager: Mrs Karen Innes; Lay representative: Mrs Heather Goodare.

18.3 IST-3 management group

Professor Peter Sandercock (Chairman and co-ordinator for trial centres in Europe and North America), Ms Karen Innes (Trial Manager), Professor Joanna Wardlaw (Neuroradiology), Professor Martin Dennis (Stroke Services). Ms Ashma Krishnan (Trial Statistician), Professor Richard Lindley (Co-ordinator for centres in Australasia)

18.4 International Advisory Board

National Co-ordinator from each country, Professor Gary Ford (UK Thrombolysis Advisor), Professor Stephen Davis (EPITHET trial liaison), Professor Werner Hacke (ECASS 3 Liaison), Professor Geoff Donnan (EXTEND Trial Liaison).

18.5 Independent Data Monitoring Committee (DMC)

Professor Rory Collins, Oxford University, UK (Chairman), Professor Philip Bath (Nottingham University), Professor Richard Gray (University of Birmingham), Dr Salim Yusuf (McMaster University, Canada), Professor Robert Hart (San Antonio, USA)

18.6 Independent events adjudicator

Dr Keith Muir, Institute of Neurological Sciences, Glasgow.

18.7 CT/MR image reading panel

The international panel of experts include: Professor Joanna Wardlaw (University of Edinburgh, IST-3 Imaging PI and Steering Committee); Dr Andrew Farrall (University of Edinburgh); Professor Rüdiger von Kummer (University of Dresden); Dr Anders von Heijne (Danderyd Hospital, Karolinksa Institute, Stockholm); Dr Nicholas Brady (Middlesbrough); Dr Lesley Cala (University of Western Australia); Professor Andre Peeters (University Hospital St Luc, Belgium); Professor Thierry Moulin (Hopital Jean Munjoz, Besancon); Associate Professor Michael Hill (University of Calgary); Dr Zoe Morris (University of Edinburgh); Dr Gillian Potter (University of Edinburgh).

19. SPONSORSHIP

The University of Edinburgh and the Lothian Health Board act as joint sponsors for the study and hold the Clinical Trial Authorisation.

Sponsor Contact Details:

**University of Edinburgh & Lothian Health Board NHS Trust
ACCORD
R & D Management Suite
The Queen's Medical Research Institute
47 Little France Crescent
Edinburgh
EH16 4TJ**

20. REFERENCES

1. Murray CJL, Lopez AD. The Global Burden of Disease: a comprehensive assessment of mortality and disability from diseases, injuries and risk factors in 1990 and projected to 2020. Boston: Harvard University Press; 1996.
2. Asplund, K. Stroke in Europe: widening gap between East and West. Cerebrovascular Diseases 1996; 6: 3-6.
3. Chen ZM, Sandercock P, Pan HC, Counsell C , Collins R, Liu LS et al. On behalf of the CAST and IST collaborative groups. Indications for early aspirin use in acute ischemic stroke : A combined analysis of 40 000 randomized patients from the Chinese acute stroke trial and the international stroke trial. Stroke 2000;31:1240-9.
4. The National Institute of Stroke and Neurological Disorders rt-PA Stroke Study Group. Tissue Plasminogen Acivator for Acute Ischemic Stroke. NEJM 1995;333(24):1581-7.
5. Hacke W, Kaste M, Fieschi C, Danilo T, Lesaffre E, von Kummer R et al. Intravenous thrombolysis with recombinant tissue plasminogen activator for acute hemispheric stroke. JAMA 1995;274(13):1017-25.
6. Hacke W, Kaste M, Fieschi C, von Kummer R, Davalos A, Meier D et al. Randomised double-blind placebo-controlled trial of thrombolytic therapy with intravenous alteplase in acute ischaemic stroke (ECASS II). Lancet 1998;352:1245-51.
7. Clark WM, Wissoman S, Hamandas JH, Albers GW, Madden KP, Hamilton S et al. Recombinant tissue-type plasminogen activator (Alteplase) for ischemic stroke 3 to 5 hours after symptom onset. The ATLANTIS Study: a randomized controlled trial. JAMA 1999;282:2019-26.
8. Albers, G. W., Clark, W. M., Madden, K. P., and Hamilton, S. A. The ATLANTIS T-PA Acute Stroke Trial: results for patients treated within three hours of stroke onset. Stroke 2000; 31(1): 307.
9. Wardlaw, J. M., del Zoppo, G., Yamaguchi , T., and Berge, E. Thrombolysis for acute ischaemic stroke (Cochrane Review). In: The Cochrane Library Issue 3. 2003. Oxford, Update Software.
10. Albers, G. W., Clark, W. M., and for the ATLANTIS Study Investigators. The ATLANTIS rt-PA (Alteplase) acute stroke trial: final results. Cerebrovascular Diseases 1999; 9 (Suppl 1): 126.
11. Donnan, G. and Davis, S. M. When is enough enough? Stroke 2001: 32, 2710-2711.
12. Lindley, R. Further Randomized Controlled Trials of Tissue Plasminogen Activator Within 3 Hours Are Required. Stroke 2001: 32, 2708-2709.
13. Lyden, P. Further randomized controlled trials of tPA within 3 hours are required-NOT. Stroke 2001: 32: 2708-2709.
14. Reed SD, Cramer SC, Blough DK, Meyer K, Jarvik JJ. Treatment with tissue plasminogen activator and inpatient mortality rates for patients with ischemic stroke treated in community hospitals. Stroke 2001;32:1832-40.
15. Johnston S, Fung L, Gillum L, Smith W, Brass L, Lichtman J et al. Utilization of intravenous tissue-type plasminogen activator for ischemic stroke at Academic centers. The influence of ethnicity. Stroke 2001;32:1061-8.
16. Horowitz SH. Thrombolytic therapy in acute stroke: neurologists, get off your hands! [see comments]. Archives of Neurology 1998;55(2):155-7.
17. Kaste, M. Approval of alteplase in Europe: will it change stroke management? Lancet Neurology 2003; 2: 207-208.
18. Canadian Association of Emergency Physicians Committee on Thrombolytic Therapy for Acute Ischemic Stroke. Thrombolytic therapy for acute ischemic stroke. Canadian Journal of Emergency Medicine 2001;3:8-12.
19. Goyal, D. G., Li, J., Mann, J., and Schriger, D. L. American Academy of Emergency Physicians position statement on the use of intravenous thrombolytic therapy in the treatment of stroke. http://www.aaem.org/positionstatements/thrombolytictherapy.html . 2003.

20. Caplan, L., Mohr, J. P., Kistler, J. P., Koroshetz, W., and Grotta, J. Clinical Debate: Should thrombolytic therapy be the first-line treatment for acute ischaemic stroke? New England Journal of Medicine 1997; 337: 1309-1313.
21. Adams, H. P., Adams, R. J., Brott, T., del Zoppo, G., Furlan, A. J., Goldstein, L. B., Grubb, R. L., Higashida, R., Kidwell, C. S., Kwiatkowski, T., Marler, J., and Hademenos, G. J. Guidelines for the Early Management of Patients With Ischemic Stroke. A Scientific Statement From the Stroke Council of the American Stroke Association. Stroke 2003: 34; 1056-1083.
22. Sandercock, P., Berge, E., Dennis, M., Hand, P., Kwan, J., Lewis, S., Neilson, A. R., Lindley, R., Thomas, B., and Wardlaw, J. M. A systematic review of the effectiveness, cost-effectiveness and barriers to the implementation of thrombolytic and neuroprotective treatment in the NHS. Health Technology Assessment 2002: 6(26).
23. Intercollegiate Working Party for Stroke. National Clinical Guidelines for Stroke Update 2002. 2002. London, Royal College of Physicians.
24. Langhorne, P. and Pollock, A. What are the components of effective stroke unit care? Age & Ageing 2002: 31(5): 365-371.
25. Leonardi-Bee, J., Bath, P. M., Phillips, S., Sandercock, P., and International Stroke Trial Collaborative Group. Blood Pressure and Clinical Outcomes in the International Stroke Trial. Stroke 2002; 33, 1315-1320.
26. Tanne, D., Kasner, E. S., Demchuk, A. M., Koren-Morag, N., Hanson, S., Grond, M., Levine, S., and the Multicenter rt-PA Stroke Survey Group. Markers of increased risk of intracerebral hemorrhage after intravenous recombinant tissue plasminogen activator therapy for acute ischemic stroke in clinical practice. The multicenter rt-PA acute stroke survey. Circulation 2002: 105, 1679-1685.
27. Ciccone, A. Consent to thrombolysis in acute ischaemic stroke: from trial to practice. Lancet Neurology 2003: 2, 375-378.
28. Koops, L. and Lindley, R. Thrombolysis for acute ischaemic stroke: consumer involvement in the design of new randomised controlled trial. British Medical Journal 2002: 325, 415-417.
29. Taves, DR. Minimization: a new method for assigning patients to treatment and control groups. Clinical Pharmacology and therapeutics. 194; 15: 443-453.
30. Counsell, C., Dennis, M., McDowall, M., and Warlow, C. Predicting outcome after acute and subacute stroke. Development and validation of new prognostic models. Stroke 2002; 33: 1041-1047.
31. Brott, T., Lu, M., Kothari, R., Fagan, S., Frankel, M., and Grotta, J. Hypertension and its treatment in the NINDS rt-PA Stroke Trial. Stroke 1998: 29, 1504-1509.
32. Blood pressure in Acute Stroke Collaboration. Vasoactive drugs for acute stroke. In Cochrane Library 2003, Issue 2 Oxford: Update Software.
33. Bath, P. M., Chalmers, J., Powers, W., Beilin, L. J., Davis, S., Lenfant, C., Mancia, C., Neal, B., Whitworth, J., and International Society of Hypertension Writing Group. International Society of Hypertension (ISH): statement on the management of blood pressure in acute stroke. Journal of Hypertension 2003: 21(4): 665-672.
34. Ludlam, C., Bennett, B., Fox, K. A., Lowe, G., and Reid, A. Guidelines for the use of thrombolytic therapy. Haemostasis and Thrombosis Task Force of the British Committee for standards in Haematology. Blood Coagulation & Fibrinolysis 1995; 6(3): 273-285.
35. Von Clauss, A. Gerinnungsphysiologische Schnellmethode zur Bestimmung des Fibrinogens. Acta Haematol 1957;17: 237-246.
36. Hill, M. D., Barber, P. A., Takahashi, J., Demchuk, A. M., Feasby, T. E, and Buchan, A. M. Anaphylactoid reactions and angioedema during alteplase treatment of acute stroke. CMAJ 2000: 162; 1281-1284.
37. Multicentre Acute Stroke Trial Italy (MAST-I) Group. Randomised controlled trial of streptokinase, aspirin, & combination of both in treatment of acute ischaemic stroke . Lancet 1995;346:1509-14.

38. Antithrombotic Trialists' Collaboration. Collaborative meta-analysis of antiplatelet therapy for prevention of death, myocardial infarction and stroke in high-risk patients. BMJ 2002;324:71-86.
39. Bamford, J. M., Sandercock, P., Warlow, C., and Slattery, J. Interobserver Agreement for the Assessment of Handicap in Stroke Patients. Stroke 1989: 20, 828-828.
40. Lindley, R., Waddell, F., Livingstone, M., Warlow, C., Dennis, M., and Sandercock, P. Can simple questions assess outcome after stroke? Cerebrovasc Dis 1994; 4: 314-324.
41. Dorman P, Dennis MS, Sandercock P. Are the modified "simple questions" a valid and reliable measure of health related quality of life after stroke? JNNP 2000;69:487-93.
42. Dorman, P., Slattery, J., Farrell, B., Dennis, M., Sandercock, P., and the United Kingdom collaborators in the International Stroke Trial. A randomised comparison of the EuroQol and Short Form-36 after stroke. British Medical Journal 1997: 315, 461-461.
43. Bamford, J. M., Sandercock, P., Dennis, M., Burn, J., and Warlow, C. Classification and natural history of clinically identifiable subtypes of cerebral infarction. Lancet 1991: 337;1521-1526.
44. Peto, R., Pike, M., Armitage, P., Breslow, N., Cox, D., and Howard, S. Design and analysis of randomised clinical trials requiring prolonged observation of each patient. II. analysis and examples. British Journal of Cancer 1977; 35: 1-39.
45. MRC Clinical Trials Series: MRC Guidelines for Good Clinical Practice in Clinical Trials. London: Medical Research Council. 1998.
46. Hacke W, Donnan G, Fieschi C, Kaste M, von Kummer R, Broderick JP, Brott T, Frankel M, Grotta J, Haley E Jr, Kwiatkowski T, Levine S, Lewandowski C, Lu M, Lyden P, Marler J, Patel S, Tilley B, Albers G, Bluhmki E, Wilhelm M, Hamilton S: Association of outcome with early stroke treatment: pooled analysis of ATLANTIS, ECASS, and NINDS rt-PA stroke trials. Lancet 363:768-74. 2004 March 6.

APPENDIX 1: KEY CONTACTS

Chief Investigator:

Peter A. G. Sandercock
Professor
University of Edinburgh
Department of Clinical Neurosciences
Bramwell Dott Building
Western General Hospital
Crewe Road
Edinburgh
EH4 2XU
0131 537 2928
Peter.sandercock@ed.ac.uk

Trial Co-ordinator:

Karen Innes
University of Edinburgh
Department of Clinical Neurosciences
Bramwell Dott Building
Western General Hospital
Crewe Road
Edinburgh
EH4 2XU
0131 537 2793
Karen.innes@ed.ac.uk
