# Supplementary material for: Update on the third international stroke trial (IST-3) of thrombolysis for acute ischaemic stroke and baseline features of the 3035 patients recruited
Source: Trials. 2011 Nov 30;12:252. doi: 10.1186/1745-6215-12-252 (PMC3286387; doi:10.1186/1745-6215-12-252)
Supplement: Additional file 2 — IST3 collabs list 141011. List of all collaborating centres and investigators with the number of patients recruited by each centre. [file 1745-6215-12-252-S2.doc]

**List of participating hospitals in each country. Figures in parentheses are the number of patients recruited in the country or by the centre.**

***UK*** *(1447)*-Royal Hallamshire Hospital (118): G Venables, C Blank, H Bowler, C Doyle, K Endean, K Harkness, E Parker, M Randall. University Hospital of North Staffordshire (97): C Roffe, N Ahmad, A Arora, S Brammer, J Chembala, B Davies, S Ellis, E Epstein, K Finney, C Jackson, C Jadun, R Kinston, H Maguire, I Memon, I Natarajan, M Poulson, R Sanyal, S Sills, A Vreeburg, E Ward. Western General Hospital (95): P Sandercock, R Al-Shahi Salman, R Davenport, M Dennis, P Hand, S Hart, I Kane, S Keir, M MacLeod, L McKinlay, H Milligan, E Sandeman, J Stone, C Sudlow, P Taylor, J Wardlaw, C Warlow, W Whiteley, A Williams. The National Hospital for Neurology & Neurosurgery (84): M Brown, B Athwal, V Bassan, N Bhupathiraju, J Bowler, C Davie, D Doig, R Erande, S Gilbert, L Ginsberg, R Greenwood, S Gregoire, N Harding, N Losseff, R Luder, N Passeron, R Perry, P Rayson, R Simister, S Stone, D Werring. Arrowe Park Hospital (83): J Barrett, H Aitken, S Cherian, R Davis, S Downham, L Godd, V Gott, D Jose, V Little, D Lowe, L Luxford, M McGrory, P Owings, N Price, J Richards, G Sangster, J Sherlock, S Vargese, I Wakefield, P Weir. Southend University Hospital (77): P Guyler, T Attygale, S Chandler, L Coward, S Feasey, C Khuoge, T Loganathan, S Martin, A O'Brien, D Sinha, V Thompson, S Tysoe, R Walsh. Norfolk and Norwich University Hospital (67): K Metcalf, J Cochius, R Fulcher, N Gange, C Green, J Jagger, M Lee, P Myint, J Potter, G Ravenhill, S Shields, N Shinh, T Staunton, E Thomas, W Woodward, P Worth, N Wyatt. Nottingham City Hospital (63): W Sunman, P Bath, P Berman, J Clarke, C Gaynor, F Hammonds, R Harwood, K Mitchell, S Munshi, S Pacey, A Shetty, N Sprigg, H Stear, G Subramanian, A Wills. Guy's & St.Thomas Hospital (60): A Rudd, H Audebert, A Bhalla, J Birns, R Chowdhury, G Cluckie, I Davies, C Gibbs, P Holmes, N Mitchell, F Schiavone, E White, M Yeung. Darlington and Bishop Auckland Hospitals (56): A Mehrzad, V Baliga, E Brown, L Burnside, B Esisi, J Kent, P Orr, D Stead, E Wayman. University Hospital Aintree (46): R Durairaj, C Cullen, R Kumar, H Martin, D McDowell, A Sharma, V Sutton, R White. University Hospital of Wales (46): T Hughes, K Ali, J Anderson, K Baker, K Bethune, K Bethune, M Booth, M Cossburn, S Halpin, M Hourihan, E Marsh, K Peall, R Powell, H Shetty, M Wardle, M Williams. Derby Royal Hospital (37): K Muhiddin, J Beavan, M Clarke, R Donneley, S Elliott, P Fox, P Gorman, M Harper, M Mangoyana, I Memon, L Mills, L Wright. Addenbrookes Hospital (34): L Warburton, J Baron, P Barry, D Day, T Harold, P Martin, J Mitchell, E O'Brien, J Rycarte, M Turnham. St George's Healthcare NHS Trust (34): G Cloud, L Choy, B Clarke, C Griffin, O Halse, I Jones, F Kennedy, U Khan, R Lewis, A Loosemore, C Lovelock, H Markus, B Moynihan, J O'Reilly, O Paul, A Pereira, M Punter, P Rich, D Rolfe, F Schiavone. Royal Devon & Exeter Hospital (Wonford) (30): M James, J Bell, A Bowring, L Boxall, J Cageao, H Eastwood, S Elyas, F Hall, S Harries, A Hemsley, S Jackson, S Keenan, P Mudd, A Sekhar, D Strain, J Sword, N Wedge. Aberdeen Royal Infirmary (26): M MacLeod, M Bruce, A Joyson, M Kemp, K McMullan, J Reid, O Robb, J Webster, S Wilkinson. Hammersmith Hospitals & Imperial College (24): P Sharma, P Bentley, H Jenkins, A Kar, T Sachs. Northwick Park Hospital (20): D Cohen, R Bathula, J Devine, M Mpelembue. William Harvey Hospital (20): D Hargroves, I Balogun, L Cowie, A Maidment, D Rand, J Rowe, H Rudenko, D Smithard, L Wray. Scarborough Hospital (17): J Paterson, J Brown, J Hampton, S Jamieson, R Rose, A Volans. Countess of Chester Hospital NHS Foundation Trust (17): K Chatterjee, G Abbott, R Brookes, C Castle, C Kelly, S Leason, A Nallasivan, A Sen. Watford General Hospital (17): D Collas, M Cottle, N Damani, P Jacob, D Oza, D Werring. University Hospitals Coventry & Warwickshire NHS Trust (15): A Kenton, N Adab, L Aldridge, H Allroggen, Y Brown, R Cross, L Galvin, K Ghosh, A Grubneac, A Lindahl, H Mehta, M Pritchard, C Randall, P Ray, A Shehu, S Thelwell. Royal Bournemouth & Christchurch NHS Trust (12): D Jenkinson, J Bell, T Black, O David, J Kwan, A Orpen, C Ovington, D Tiwari, Z ud Din Babar. Leeds General Infirmary (12): A Hassan, A Bailey, J Bamford, C Bedford, R Bellfield, J Cooper, L Dunsmure, J Greig, M Keeling, L Mandizvidza, J Rankine, E Roberts, P Wanklyn, T Webb, S Williamson. York Health Services NHS Trust (12): J Coyle, S Crane, C Croser, P Duffey, R Evans, E Iveson, M Keeling, G Kitching, M Porte, C Rhymes. Queen Elizabeth Hospital (Gateshead) (12): D Barer, M Armstrong, M Bokhari, T Cassidy, B McClelland. Queen Elizabeth The Queen Mother Hospital (10): G Gunathilagan, P DOLKE, S Jain, S Jones, A Maidment, L Rosser, G Thomas, C White. Worcestershire Royal Hospital (10): P Sanmuganathan, C Scholtz, E Stratford. Blackpool Victoria Hospital (10): M O'Donnell, H Goddard, G Hoadley, J Howard, S Leach, J McIlmoyle, A Stewart, A Strain. Basildon & Thurrock University Hospitals NHS FT (9): F Huwez, P Croot, N Gadi, N Mguni, U Umasankar. Royal Infirmary of Edinburgh (8): G Mead, B Chapman, A Coull, S Hart, A Kinnear, B Morrow, F Morrow. St Mary's Hospital (8): D Ames, J Ball, S Bannerjee, J Chataway. Yeovil District Hospital (8): K Rashed, C Buckley, D Donaldson, D Hayward, C Lawson. Luton and Dunstable Hospital (8): L Sekaran, K Bharaj, F Justin, G Jutlla, D Phiri, S Sethuraman, M Tate. Solihull Hospital, Heart of England NHS Trust (8): D Sandler, P Carr, G Jones, J Lyons, K Warren. King's College Hospital (7): L Kalra, A Davis, J Jarosz, D Manawadu, L Sztriha. Doncaster Royal Infirmary (7): D Chadha, A Holford, P Willcoxson. Royal United Hospital Bath (7): L Shaw, D Button, A Cunningham, L Dow, J Dutson, T Hall, C Hardy, N Jakeman, P Kaye, B Madigan, K O'Brien, D Pressdee, M Price, L Robinson, C Taylor, D Williamson. Birmingham Heartlands Hospital (6): D Sandler, P Carr, J Lyons, J McCormack, C Stretton. University Hospital North Durham (6): P Earnshaw, E Brown, S Bruce, C Church, S Desai, B Esisi, M Myint, N Watt. Wansbeck General Hospital (6): C Price, S Elliott, H Graham, R Lakey, K Mitchelson. Bristol Royal Infirmary (6): P Murphy, L Ball, S Caine, J Dovey, J Hughes, A Steele. Stepping Hill Hospital (6): K Dizayee, A Brown, T Chattopadhyay, J Cheetham, H Cochrane, A Datta, M Datta-chaudhuri, C Fox, D Kilroy, S Krishnamoorthy, F Levy, S Metha, P Ngoma, B Venkatesh. Princess Royal Hospital Brighton & Sussex University Hospitals Trust (5): K Ali, R Gautam, N Henderson, M Jones, S Murphy, G Spurling. Belfast City Hospital (5): I Wiggam, C Boyd, K Fullerton, P Gray, M Kinnaird, S MacNair, C Morgan, M Reid, S Tauro. Royal Liverpool University Hospital (5): S Loharuka, D Balmforth, P Cox, G Fletcher, A Ledger, A Manoj, M Wilkinson. City Hospital, Sandwell & West Birmingham Hospital (5): D Nicholl, S Clegg, S Hurdowar, S Kausar, K Law, A Singal, S Sturman. Royal London Hospital (4): P Gompertz, J Evanson, A Farrell, A Petrou, K Saastamoinen, T Sachs, A Salek-Haddadi, R Yadava. Sunderland Royal Hospital (4): J O'Connell, H Brew, S Butler, S Crawford, C Gray, D Gulliver, N Majmudar, R O'Brien. Morriston Hospital (4): M Wani, L Dacey, L Davies, R Evans, D Harris, T Jones, S Storton. Royal Preston Hospital (4): S Punekar, A Ashton, S Duberley, H Emsley, C Gilmour, B Gregary, L Hough, S Philip, S Wuppalapati. The Royal Wolverhampton Hospitals NHS Trust (4): K Fotherby, P Bourke, D D'Costa, K Kauldhar, D Leung, R Lodwick, S McBride, D Morgan, M Qaiyum, G Sahota, M Srinvasan. Royal West Sussex NHS Trust, St Richard's Hospital (4): I Kane, N Chuter, L Garrad, M Hookway, S Ivatts, G Kennedy. Queen's Hospital Romford (4): K Darawil, L Al Dhahirl, S Andole, M Baig, P Dugh, K Dunne, H Kariuki, M Khan, S Rathnayaka. Ulster Hospital (3): M Power, K Dynan, J Finnerty, A Heaney, C Leonard, K McKnight, J Turkington, B Wroath. Great Western Hospital (3): B Dewan, S Cotton, M Gardiner, T Saunders, B Vincent. The Queen Elizabeth Hospital Birmingham (3): D Sims, P Guest, E Jones, J McCormack, D Nicholl, J Savanhu, R Tongue, M Willmot. Leicester General Hospital (3): D Eveson, S Dawson, M Dickens, M Fotherby, R Hunt, S Khan, T Kumar, R Marsh, A Mistri, T Robinson, J Thompson. Darent Valley Hospital, Dartford & Gravesham NHS Trust (3): P Aghoram, T Daniel, M Gatehouse, S Hussein, A Jackson, T Shanganya, E Strachan, G Tan. Nevill Hall Hospital, Aneurin Bevan Local Health (3): B Richard, S Elaine, S Hanson, S Mosely, H Reed, M Williams. Colchester Hospital University Foundation Trust (3): R Saksena, S Cook, D Demuran, M Keating, R Needle, V Paramsothy, A Sebastian, R Sivakumar, A Wright. Salford Royal Hospital Foundation NHS Trust (2): R Grue, E Barberan, C Dickson, C Douglas, J Jellicoe, T Marsden , J Priestley, E Quick, C Sherrington, A Singh, C Smith, J Stevens, P Tyrell, J Wainwright. Leicester Royal Infirmary (2): M Ardron, J Birchall. Queen Elizabeth Hospital (Kings Lynn) (2): R Shekhar, C Barsted, S Coleman, S Fletcher, J Graham. John Radcliffe Hospital (2): A Buchan, J Hinkle, J Kennedy, A Manoj, M Westwood. Derriford Hospital (2): A Mohd Nor, S Allder, B Hyams, A Pace. West Cumberland Hospital (1): E Orugun, C Brewer, L Huntley, R Jolly, C Summers. Sandwell General Hospital (1): K Sharobeem, J Khaira, J Leahy, E Linehan, G Moore, J Rizkalla, J Wilkinson. Torbay Hospital (1): D Kelly, C Hilaire. Warrington & Halton Hospitals NHS Foundation Trust (1): O Otaiku, L Connell, G Delaney-Sagar, G James, L Lomax, D Matthew, J Simpson, H Whittle. Medway Maritime Hospital (1): S Sanmuganathan, S Burrows, A Mahmood. Southampton General Hospital (1): G Durward, S Barker, J Cantle, P Crawford, S Evans, V Pressly, N Weir. Victoria Hospital (1): V Cvoro, K McCormick. 
***Poland*** *(347)*-Institute of Psychiatry & Neurology (190): A Czlonkowska, J Bembenek, M Bilik, G Chabik, W Czepiel, J Dzierka, M Gluszkiewicz, K Grabska, B Janus-Laszuk, J Jedrzejewska, A Kobayashi, T Litwin, A Oskedra, A Piorkowska, M Skowronska, A Sliwinska, U Stepien. SPZZOZ w Sandomierzu (43): P Sobolewski, A Gajewska, M Grzesik, R Hatalska-Zerebiec, I Labudzka, B Loch, A Medrykowska, M Sledzinska, A Sobota, W Szczuchniak, G Wolak, I Zdyb. Medical University of Gdansk (35): W Nyka, D Gasecki, K Chwojnicki, A Gojska, B Karaszewski, G Kozera, M Kwarciany, M Nowak, M Swierkocka-Miastkowska , S Szczyrba, M Wisniewska, E Wnorowska. 1st Dept, Institute of Psychiatry & Neurology (25): P Richter, A Bochynska, M Chahwan, A Graban, R Rola. Military Medical Institute (24): A Stepien, B Brodacki, M Grotowska, J Kotowicz, J Staszewski, J Swistak, S Zaloga. Szpital Powiatowy (14): J Stoiñski, K Czajkowaka-Fornal, P Czubak, A Kaczor, J Kraska, E Nowakowska-Sledz, J Ozdoba-Rot, E Zawadzka. Szpital Specjalistyczny w Konskich (8): M Fudala, D Adamczyk, W Brola, I Guldzinska, K Kaluzny, M Kucharska-Lipowska, M Mosiolek, M Polewczyk, M Ziomek. Central University Hospital (7): G Opala, M Arkuszewski, M Kudlacik, P Malgorzata, M Swiat. SPSK im. Prof. W. Orlowskiego CMKP (1): U Fiszer, M Lenska-Mieciek. 
*Italy (326)*-Ospedale Di Citta' di Castello (62): S Cenciarelli, A Barilaro, R Condurso, F Coppola, S Dioguardi, E Gallinella, A Mattioni, C Menichetti, S Ricci. Nuovo Ospedale Civile "S.Agostino-Estense" (40): F Casoni, M Bacchelli, M Cavazzuti, M Malagoli, A Zini. Ospedale Beato Giacomo Villa - Citta' della Pieve (36): G Benemio, M Celani, R Allegrucci, V Bondo, S Cupella, L Guerra, S Guerrieri, C Ottaviani, E Righetti, C Rossi, N Sacchi, M Scucchi, V Stefanini. Ospedale Di Branca (36): T Mazzoli, A Bigaroni, L Greco, R Paris, P Parise, S Ricci. A.O. Niguarda Ca'Granda (25): A Ciccone, L Basso, R Causarano, P Doneda, E Ferrante, A Gatti, A Guccione, A Gullo, F Imbesi, S Jann, R Marazzi, E Moro, C Motto, D Parodi , A Protti, M Riva, A Rosiello, I Santilli, R Sterzi, P Tiraboschi, G Venturelli. R.Guzzardi Hospital - Vittoria (RG) (19): F Iemolo, R Campagna, G Campagnolo, A Carnemolla, N D'Apico, G D'Asta, S Giannarita, A Giordano, E Sanzar. Ospedale Regionale di Aosta (17): E Bottacchi, S Cordera, G Corso, M Di Giovanni, G Giardini, C Lia, T Meloni, M Pesenti Campagnoni, P Tosi. Ospedale Sacro Cuore - Negrar Verona (12): A Adami, G Rossato, T Zuppini. Ospedale Maggiore - Bologna (12): G Procaccianti, T Sacquegna. Universita degli Studi di Genova (11): C Gandolfo, M Balestrino, C Bruno, L Castellan, M Del Sette, A Ferrari, C Finocchi, N Reale, D Rizzi. Ospedale Civlle S.Andrea (9): M Del Sette, L Benedetti, C Capellini, E Carabelli, E Cibei, M Godani, G Guariglia, E Landini, A Mannironi, B Nucciarone, S Parodi, S Tonelli, E Traverso, D Zito. Ospedale S. Giovanni Battista - Foligno (8): P Brustenghi, F Corea, O Flamini, S Lolli, G Pelliccia, R Ricci, S Stefanucci, M Zampolini. Ospedale a Vibo Valentia (8): D Consoli, F Galati, P Postorino. Azienda Ospedaliero-Universitaria "Ospedali Riuniti" di Foggia (8): G Rinaldi, E Carapelle, G Grilli, M Guido, L Specchio. Ospedale Valduce di Como (8): N Checcarelli, G Borin, L Chiveri, R Clerici, E Corengia, L Gandola, P Garavaglia, M Guidotti, A Martegani, M Mauri, F Muscia, F Raudino. Ospedale di Cattinara - Trieste (4): F Chiodo Grandi, A Bratina, N Carraro, M Gaio, A Granato, N Koscica, M Naccarato, V Sarra, P Schincariol, C Vilotti, Z Zugna. Clinica Dr Pederzoli Spa (4): D Idone, C Bonato, E De Angelis, A Forgione, M Gambera, F Recchia, S Tamburin, P Tinazzi Martini, G Zanette. Ospedale Civile San Matteo Degli Infermi - Spoleto (2): S Grasselli. Ospedale Silvestrini - Perugia (2): G Agnelli, A Andrea, A Billecia, V Caso, V Casso, R Fabiola, P Fanelli, M Paciaroni, B Sergio, M Vemti. Mater Salutis Hospital, Legnago VR (2): M Silvestri, L Altarini, A Bonfante, M Bonornetti, B Costa, N D'Attoma, N Deluca, F Frattini, R Niego, D Rafaele, V Ravenna, M Turazzini. Ospedale Guglielmo da Saliceto - Piacenza (1): S Cammarata. 
***Sweden*** *(297)*-Uppsala University Hospital (100): E Lundström, L Jonsson , U Söderström, A Terént. Danderyd Hospital (46): V Murray, A Alvelius, M Arbin von, I Dalenbring, Å Doverhall, Å Franzén-Dahlin, N Greilert, M Hallberg, A Heijne von, E Isaksson, H Kumpulainen, A Laska, A Lundström, C Martin, J Muhrbeck, E Näslund, N Ringart, E Rooth, R Undén, P Waldenström. Hassleholm Hospital (29): M Esbjornsson, M Petranek. Karnsjukhuset (25): B Cederin, E Bertholds, A Elgåsen, T Johansson, B Witteborn. Koping Hospital (20): M Kwiatkowska, E Gustafsson, T Noren, J Saaf. Mora Hospital (17): J Teichert, M Bertilsson, S Nilsson, S Oestberg. LidkopingHospital (11): L Welin, K Fredricson, L Pehn. Falu Hospital (11): J Hambraeus, I Lonn. Capio S: tGoran Hospital (9): B Hojeberg, A Adolfsson, M Anzen. Vastervik Hospital (5): T Wallen, R Schloenzig, P Söderström, A Wennerberg. University Hospital MAS (5): F Buchwald, K Abul-Kasim, A Berkeskold, J Petersson, E Poromaa. University Hospital of Northern Sweden (4): P Wester, R Backlund, A Sjöström. Helsingborgs lasarett (4): B Hedström , E Campbell, K Johnsson, B Karlsson, N Lekokotla, C Lundahl, A Risedal, P Sandgren, A Svensson. Visby Hospital (4): S Bysell, E Smedberg, A Vestberg Bysell. Sundsvall Hospital (3): V Sjögren, B Högvall. University Hospital Lund (2): G Andsberg, T Cronberg, A Lindgren. Vasteras Hospital (1): H Wannberg, F Ax, L Nyren. Karlstad Central Hospital (1): J Sanner, H Andersson, F Andler, S Holmgård, R Johansson, I Magnussan, K Nilsson, J Rådberg. 
*Norway (204)*-University Hospital Trondheim (69): B Indredavik, H Elleksaer, A Ostvik, G Rohweder, D Steckhan, J Storvold. Ulleval Sykehus (66): E Berge, Y Ronning, R Aakvik, K Bruins Slot, G Knutsen, M Moxness, R Pettersen, T Wyller. University Hospital North Norway (23): C Wahl, O Iversen, S Johnsen, B Norderhus, L Steffensen, E Stensland. Kongsvinger Sykehus (13): T Asak, J Aaseth, T Rotnes, J Sparby, S Wetterhus. Sykehuset Levanger (9): H Hallan, A Aarolal, T Graven, H Hansbakk Skjetne, B Klykken, K Lindqvist, A Tommy. University Hospital Northern - Norway (8): T Engstad, M Antonsen, R Bajic, W Fønnebø, S Hykkerud, I Lyngmo, A Nyrnes, S Rogne, S Sparr. Harstad Sykehus (7): O Kildahl-Andersen, K Pedersen, H Ulrichsen. Alesund Sjukehus (4): O Skogen, I Alnes, R Hukari, I Seljeseth, P Vadset. Medisinsk Avdeling, Asher and Baerum Hospital (2): G Knutsen, b Fure, H Ihle-Hansen, N Johnsen, L Kornberg. Sykehuset Namsos (2): S Schuler, M Heibert. Volda sjukehus (1): M Lillebo, O Aasen, I Eskeland, T Hamre, S Hareide, H Helset, K Kolnes, B Lodemel, H Ose Velle, S Reite, E Velle. 
***Australia*** *(179)*-Nambour General Hospital (51): R Grimley, E Ahern, C Cocks, M Courtney, R Devin, J Endacott , C Fawcett , V Harrington, C Johnston, M Koltermann, S Murray, K Ng, G Styles, A Tampiyappa. John Hunter Hospital (29): C Levi, K Chung, L Dark, M Evans, Y Gawarikar, E Kerr, A Loiselle, F Miteff, A Moore, W O'Brien, M Parsons, D Quain, A Royan, M Russell, N Spratt. Gosford Hospital (24): J Sturm, D Crimmins, D Griffiths, P Kavelieros, J Kinsella, A Malhotra, B O'Brien, A Schutz, M Webb, S Whyte, V Zenteno. Westmead Hospital (16): R Lindley, A Bleasel, N Cordato, A Duggins, V Fung, L Gomes, N Ingham, J Ip, P Landau, J Morris, S Vucic. Royal Perth Hospital (15): G Hankey, A Claxton, N Lillywhite. The Canberra Hospital (12): C Lueck, C Andrews, G Danta, C Das, I Harvey, A Hughes, C McColl, A Oon, R Tuck. Royal Brisbane and Women's Hospital (10): S Read, M Badve , M Broad, G Cadigan, H Cavanagh, J Chalk, D Copsinis, K Etherington, R Henderson, R Hull, J O'Sullivan, J Pandian, L Ross-Lee, M Roxas, N Sheikh, G Skinner, A Wong. Austin Health - Repatriation Campus (8): H Dewey, A Brodtmann, G Donnan, A Hughes, M Karonen, H Ma, T Mulcahy, S Petrolo, L Walker, D Young, J Zavala. Nepean Hospital (7): M Thieben, C Harris, M Krause, S Lane, H Park, M Shaffi, J Wood. Box Hill Hospital (7): C Bladin, A Buckland, K Coughlan, B Coulton, A Gilligan, P Lee, S Mullen, Z Ross, P Sien Loh, C Szoeke. 
***Portugal*** *(82)*-UAVC. Centro Hospitalar de Trás-os-Montes e Alto Douro (42): M Silva, F Afonso, J Gabriel, P Guimarães, A Velon. Hospital Pero da Covilhã (19): M Castelo- Branco, F Alvarez, V Branco, C Coxo, P Goulao, D Leal, S Morgado, R Oliveira, F Paiva, A Rodrigues, M Simoes. Hospital de Santo António (12): G Lopes, T Almeida, M Cardoso, J Chaves, C Correia, M Correia, J Damásio, R Felgueiras, J Pereira, A Tuna. Hospital S.Marcos (9): C Ferreira, E Lourenco, A Machado, R Mare, J Rocha. 
***Belgium*** *(73)*-Cliniques Universitaires St Luc (73): A Peeters. 
***Austria*** *(46)*-Landesklinikum Donauregion Tulln (34): K Matz, M Brainin, G Funk, V Reiner-Deitemyer. Krankenhaus Der Barmherzigen Bruder Wien (9): J Ferrari, A Flamm-Horak, G Gruber, R Rattinger. Krankenhaus Göttlicher Heiland (3): W Muellbacher, D Doppelbauer, R Kalchmayr, W Schima, T Wieser, M Zart. 
***Switzerland*** *(23)*-Universitatsspital Basel (22): P Lyrer, L Bonati, S Engelter, F Fluri, S Muller, E Radue, A Tiemessen, L Walz, F Weisskopf, S Wetzel. Universitätsspital Zürich (1): A Luft, D Fetz, B Hertler, A Pangalu. 
***Canada*** *(8)*-QEII Health Sciences Centre (8): G Gubitz, P Boulton, J Jarrett, J Moeller, S Phillips. 
***Mexico*** *(3)*-Instituto Nacional de Neurologia y Neurojrugia MVS (3): A Arauz, L Bermudez, J Calleja, R Garcia.
